# A systematic review and meta-analysis of the kynurenine pathway of tryptophan metabolism in rheumatic diseases

**DOI:** 10.3389/fimmu.2023.1257159

**Published:** 2023-10-23

**Authors:** Arduino A. Mangoni, Angelo Zinellu

**Affiliations:** ^1^ Discipline of Clinical Pharmacology, College of Medicine and Public Health, Flinders University, Adelaide, SA, Australia; ^2^ Department of Clinical Pharmacology, Flinders Medical Centre, Southern Adelaide Local Health Network, Adelaide, SA, Australia; ^3^ Department of Biomedical Sciences, University of Sassari, Sassari, Italy

**Keywords:** tryptophan, kynurenine, kynurenine to tryptophan ratio, 3-hydroxykynurenine, quinolinic acid, rheumatic diseases, inflammation, biomarkers

## Abstract

There is an increasing interest in the pathophysiological role of the kynurenine pathway of tryptophan metabolism in the regulation of immune function and inflammation. We sought to address the link between this pathway and the presence rheumatic diseases (RD) by conducting a systematic review and meta-analysis of studies reporting the plasma or serum concentrations of tryptophan, kynurenine, and other relevant metabolites in RD patients and healthy controls. We searched electronic databases for relevant articles published between inception and the 30^th^ of June 2023. Risk of bias and certainty of evidence were assessed using the Joanna Briggs Institute Critical Appraisal Checklist and the Grades of Recommendation, Assessment, Development and Evaluation Working Group system. In 24 studies selected for analysis, compared to controls, RD patients had significantly lower tryptophan (standard mean difference, SMD= -0.71, 95% CI -1.03 to -0.39, p<0.001; I^2^ = 93.6%, p<0.001; low certainty of evidence), and higher kynurenine (SMD=0.69, 95% CI 0.35 to 1.02, p<0.001; I^2^ = 93.2%, p<0.001; low certainty), kynurenine to tryptophan ratios (SMD=0.88, 95% CI 0.55 to 1.21, p<0.001; I^2^ = 92.9%, p<0.001; moderate certainty), 3-hydroxykynurenine (SMD=0.74, 95% CI 0.30 to 1.18, p=0.001; I^2^ = 87.7%, p<0.001; extremely low certainty), and quinolinic acid concentrations (SMD=0.71, 95% CI 0.31 to 1.11, p<0.001; I^2^ = 88.1%, p<0.001; extremely low certainty). By contrast, there were non-significant between-group differences in kynurenic acid, 3-hydroxyanthranilic acid, kynurenic acid to kynurenine ratio, or quinolinic acid to kynurenine acid ratio. In meta-regression, the SMD of tryptophan, kynurenine, and kynurenine to tryptophan ratio were not associated with age, publication year, sample size, RD duration, C-reactive protein, or use of anti-rheumatic drugs and corticosteroids. In subgroup analysis, the SMD of tryptophan, kynurenine, and kynurenine to tryptophan ratio was significant across different types of RD, barring rheumatoid arthritis. Therefore, we have observed significant alterations in tryptophan, kynurenine, 3-hydroxykynurenine, and quinolinic acid concentrations in RD patients. Further research is warranted to determine whether these biomarkers can be useful for diagnosis and management in this patient group. (PROSPERO registration number: CRD CRD42023443718).

**Systematic review registration:**

https://www.crd.york.ac.uk/prospero, identifier CRD CRD42023443718.

## Introduction

**Figure 1:**
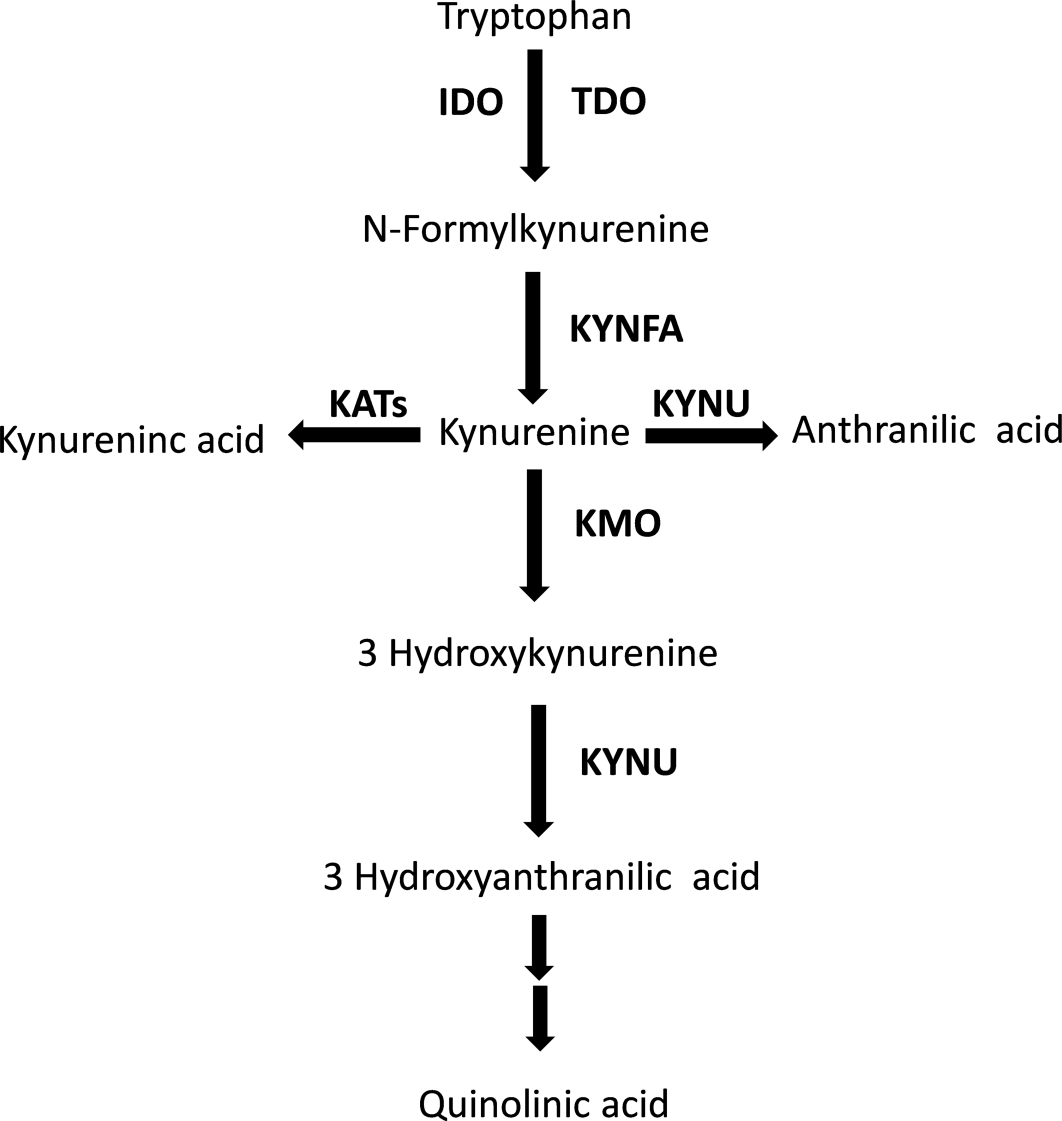
The tryptophan/kynurenine pathway. TDO, tryptophan 2,3-dioxygenase; IDO, indoleamine 2,3-dioxygenase; KYNFA, kynurenine formamidase; KATs, kynurenine aminotransferases; KMO, kynurenine 3-monooxygenase; KYNU, kynureninase.

The kynurenine pathway is responsible for approximately 95% of the metabolism of the essential amino acid tryptophan (**Figure 1**) (1). While the majority (90%) of physiological tryptophan degradation through this pathway occurs in the liver, the extra-hepatic kynurenine pathway plays a relatively greater role in states of immune activation (2). The main roles of the kynurenine pathway include the regulation of tryptophan availability for the synthesis of serotonin in the central nervous system, and the synthesis of heme, nicotinic acid, oxidized nicotinamide adenine dinucleotide (phosphate), NAD^+^(P^+^), and its reduced form NAD(P)H (3). There has been an increasing interest, particularly over the last three decades, in the biological and pathophysiological role of the kynurenine pathway. Several studies have reported that a) local and systemic alterations in kynurenine metabolites, e.g., tryptophan and kynurenine, resulting from changes in enzyme expression and/or activity suggest the presence of conditions such as cancer, immune diseases, and neuropsychiatric disorders, and b) individual kynurenine metabolites, e.g., kynurenic acid, 3-hydroxyanthranilic acid, 3-hydroxykynurenine, and quinolinic acid, can directly influence various processes, including the redox state, immune function, glutamate neurotransmission in the central nervous system, and carbohydrate metabolism (3–8).

The interplay between the kynurenine pathway, inflammation, and immunity has also stimulated research on the pathophysiological and clinical role of kynurenine metabolites in patients with rheumatic diseases (RD). RD is an umbrella term that includes various chronic systemic conditions affecting the musculoskeletal system with a predominantly autoimmune (e.g., systemic lupus erythematosus, SLE, rheumatoid arthritis, RA, Sjogren’s syndrome, SSj, systemic sclerosis, SSc, and progressive systemic sclerosis, pSS), mixed-autoimmune-autoinflammatory (e.g., psoriatic arthritis, PsA, ankylosing spondylitis, AS, axial spondylarthritis, axSpA, and Behcet’s disease, BD), or autoinflammatory component (e.g., familial Mediterranean fever, FMF) (9–11). Whilst significant progress has been made in the diagnosis and treatment of clinically overt RD, significant challenges remain with the identification of early forms of disease, which supports the search for novel RD biomarkers (12–14).

We sought to address these issues by conducting a systematic review and meta-analysis of published studies investigating the plasma or serum concentrations of metabolites within the kynurenine pathway (**Figure 1**) in patients with RD and healthy controls. We further investigated the presence of associations between the effect size of between-group differences in individual metabolites and a range of study and patient characteristics, particularly C-reactive protein, type of RD (autoimmune, mixed autoimmune-autoinflammatory, or autoinflammatory disease), and use of disease-modifying anti-rheumatic drugs (DMARDs) and corticosteroids.

## Materials and methods

### Search strategy and study selection

We systematically searched the electronic databases PubMed, Web of Science, and Scopus for relevant articles published from inception to the 30^th^ of June 2023 using the following terms and their combination: “rheumatic diseases” OR “rheumatoid arthritis” OR “psoriatic arthritis” OR “ankylosing spondylitis” OR “systemic lupus erythematosus” OR “systemic sclerosis” OR “Sjogren’s syndrome” OR “connective tissue diseases” OR “vasculitis” OR “Behçet’s disease” AND “tryptophan” OR “kynuren*” OR “anthranil*” OR “xanthurenic” OR “cinnabar*” OR “picolinic” OR “quinolinic”. Two investigators independently screened each abstract and, if relevant, the full-text articles according to the following inclusion criteria: (i) assessment of tryptophan catabolites and/or their ratio in plasma or serum, (ii) comparison of participants with RD and healthy controls aged ≥18 years in case-control studies, and (iii) full-text articles available in English language. The references of each article were also searched for additional studies. A third investigator was involved in case of disagreement.

Two investigators independently extracted the following information from the selected manuscripts and transferred them to an electronic spreadsheet for further analysis: first author, year of publication, continent where the study was conducted, number of participants, male to female ratio, tryptophan, kynurenine, kynurenine to tryptophan ratio, kynurenic acid, 3-hydroxyanthranilic acid, 3-hydroxykynurenine, quinolinic acid, kynurenine acid to kynurenine ratio, quinolinic acid to kynurenine acid ratio, C-reactive protein (CRP), disease duration, type of RD (autoimmune: SLE, RA, SSj, SSc, pSS; mixed autoimmune-autoinflammatory: PsA, AS, axSpA, and BD; autoinflammatory: FMF), use of DMARDs and corticosteroids, matrix used for analysis (serum or plasma), and analytic method used for the measurement of individual metabolites.

We assessed the risk of bias with the Joanna Briggs Institute Critical Appraisal Checklist for analytical studies (15). Studies addressing ≥75%, ≥50% and <75%, and <50% of checklist items were considered as having low, moderate, and high risk, respectively. We also assessed the certainty of evidence using the Grades of Recommendation, Assessment, Development and Evaluation (GRADE) Working Group system (16). We complied with the Preferred Reporting Items for Systematic reviews and Meta-Analyses (PRISMA) 2020 statement (17), and registered our study in the International Prospective Register of Systematic Reviews (PROSPERO registration number: CRD CRD42023443718).

### Statistical analysis

We generated forest plots of standardized mean differences (SMDs) and 95% confidence intervals (CIs) to assess for differences in serum or plasma concentrations of individual metabolites between RD patients and healthy controls (a p-value of less than 0.05 was considered statistically significant). Where necessary, means and standard deviations were extrapolated from medians and interquartile ranges or ranges, as previously reported (18, 19), or from graphs using the Graph Data Extractor software (San Diego, CA, USA). Heterogeneity of SMD across studies was assessed using the Q statistic (20, 21). The stability of the results of the meta-analysis was assessed in sensitivity analysis (22). The Begg’s and Egger’s tests and the “trim-and-fill” procedure were used to assess and eventually correct publication bias (23–25). Univariate meta-regression and subgroup analyses were conducted to investigate associations between the effect size and the following parameters: year of publication, study continent, number of participants, age, male to female ratio, CRP, disease duration, type of RD, use of DMARDs and corticosteroids, sample matrix (serum or plasma) and assay method used to measure analytes. Statistical analyses were performed using Stata 14 (Stata Corp., College Station, TX, USA).

## Results

### Screening process

**Figure 2:**
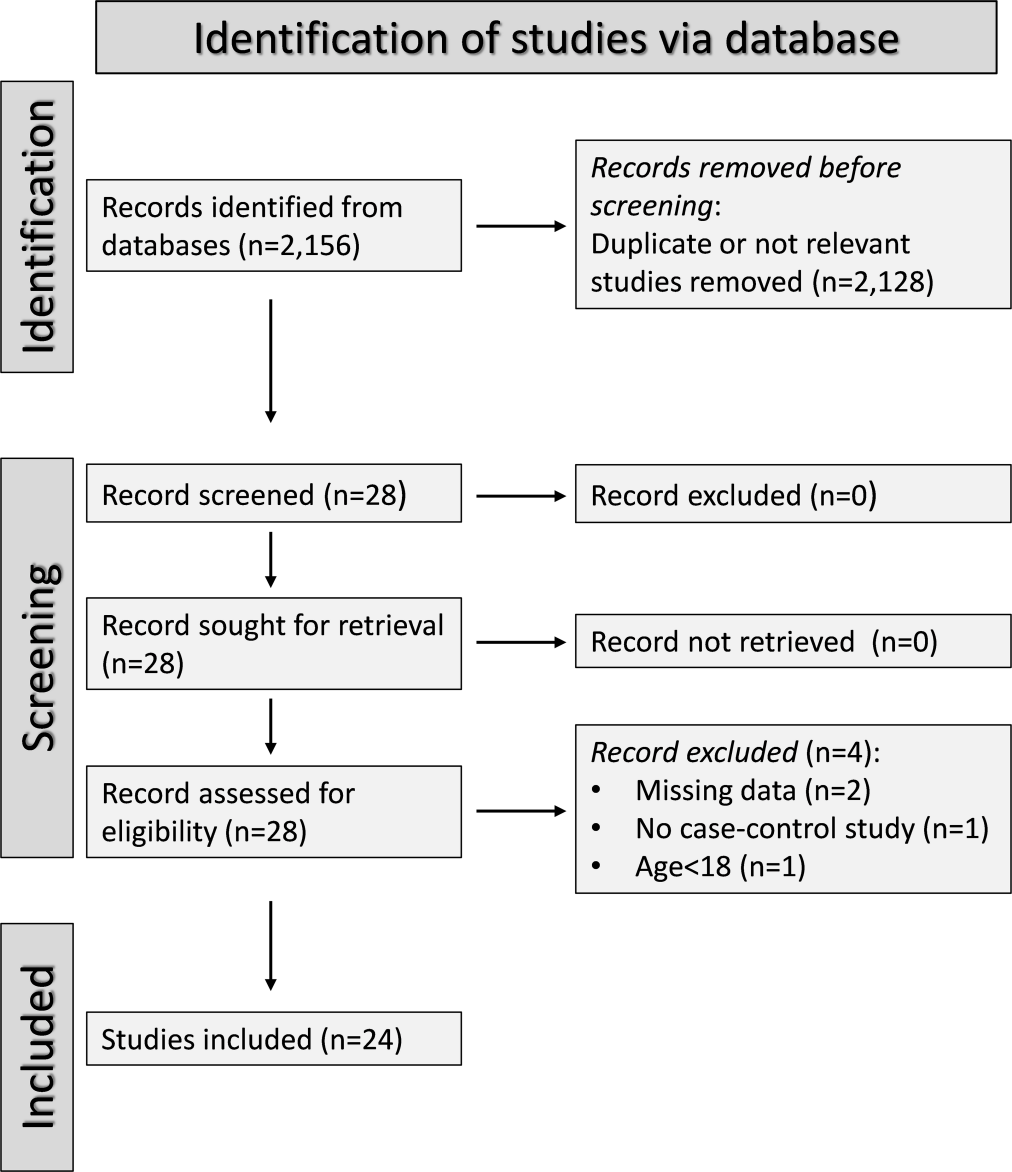
PRISMA 2020 flow chart of study selection.

A flow chart of the screening process is described in **Figure 2**. We initially identified 2,156 studies, of which 2,128 were excluded after the first screening because they were either duplicates or irrelevant. After full-text revision of the remaining 28 articles, two were further excluded because of missing data, one because it was not a case-control study, and one because it was conducted in participants <18 years of age. Thus, 24 studies were selected for analysis (**Table 1**) (26–49). The risk of bias, presented in **Supplementary Table 3**, was moderate in 14 studies (26–35, 37, 41, 44, 45, 47), and low in the remaining 10 (34, 36, 38–40, 42, 43, 46, 48, 49). The initial certainty of evidence was low for all studies (rating 2, ⊕⊕⊝⊝) given their cross-sectional design.

**Table 1:** Characteristics of the selected studies.

	Healthy controls					Patients with rheumatic diseases				
Study	n	Age (Mean or median)	M/F	Trp Kyn KynA 3HK 3HAA (Mean ± SD)	QuinA Kyn/Trp KynA/Kyn QuinA/KynA (Mean ± SD)	n	Age (Mean or median)	M/F	TRP Kyn KynA 3HK 3HAA (Mean ± SD)	QuinA Kyn/Trp KynA/Kyn QuinA/KynA (Mean ± SD)
Csipo I et al., 1995, Hungary (26)	46	NR	NR	63.1 ± 11.1 NR NR NR NR	NR NR NR NR	29	50	NR	38.2 ± 9.7 NR NR NR NR	NR NR NR NR
Widner B et al., 2000, Austria (27)	49	38	28/21	71.5 ± 12.4 1.84 ± 0.61 NR NR NR	NR 0.026 ± 0.007 NR NR	55	41	10/45	54.6 ± 13.6 2.53 ± 1.22 NR NR NR	NR 0.046 ± 0.021 NR NR
Schroecksnadel K et al., 2003, Austria (28)	20	50	NR	64.8 ± 12.8 1.98 ± 0.54 NR NR NR	NR 0.032 ± 0.005 NR NR	38	57	2/36	45.1 ± 7.1 1.9 ± 0.57 NR NR NR	NR 0.043 ± 0.009 NR NR
Pertovaara M et al., 2005, Finland (29)	309	45	170/139	79.4 ± 13.7 2.05 ± 0.48 NR NR NR	NR 0.026 ± 0.006 NR NR	103	60	7/96	74 ± 13.2 2.53 ± 1.05 NR NR NR	NR 0.034 ± 0.014 NR NR
Xiang ZY et al., 2010, China (30)	80	34	37/42	43.4 ± 4.6 1.54 ± 0.36 25.2 ± 6.5 NR NR	NR 0.037 ± 0.008 16.6 ± 3.2 NR	30	35	5/25	30.6 ± 4.1 3.72 ± 0.56 60.3 ± 5.9 NR NR	NR 0.1236 ± 0.058 16.2 ± 2.7 NR
Ozkan Y et al., 2012, Turkey (31)	20	62	4/16	42.9 ± 6.6 2.71 ± 0.65 NR NR NR	NR 0.062 ± 0.011 NR NR	32	59	5/27	43.9 ± 15.6 2.56 ± 0.56 NR NR NR	NR 0.062 ± 0.027 NR NR
Lood C et al., 2015, Sweden (32)	79	48	10/69	54.1 ± 11.4 1.85 ± 0.61 NR NR NR	NR 0.057 ± 0.0143 NR NR	148	47	22/126	49.9 ± 15.2 1.85 ± 0.66 NR NR NR	NR 0.06 ± 0.0181 NR NR
Maria NI et al., 2016, The Netherlands (33)	71	52	5/66	8,201 ± 2,163 584 ± 188 NR NR NR	NR 0.074 ± 0.0215 NR NR	124	58	8/116	6,147 ± 1,008 613 ± 219 NR NR NR	NR 0.107 ± 0.035 NR NR
Smolenska Z et al., 2016, Poland (34)	19	41	2/17	48.2 ± 26.8 NR NR NR NR	NR NR NR NR	46	42	4/42	47.7 ± 13.3 NR NR NR NR	NR NR NR NR
Åkesson K et al., 2018, Sweden (35)	30	47	NR	61.8 ± 14 0.712 ± 0.230 NR NR NR	0.38 ± 0.15 0.012 ± 0.0048 NR NR	132	48	NR	56.6 ± 23 0.966 ± 0.530 NR NR NR	0.546 ± 0.48 0.045 ± 0.02 NR NR
Urbaniak B et al., 2019, Poland (36)	51	32	10/41	50.3 ± 10.8 NR NR NR NR	NR NR NR NR	50	51	5/45	45.5 ± 10.9 NR NR NR NR	NR NR NR NR
Smolenska Z et al., 2020, Poland (37)	27	NR	7/35	40.8 ± 12.3 NR NR NR NR	NR NR NR NR	42	60	NR	32.5 ± 9.6 NR NR NR NR	NR NR NR NR
Zhou Y et al. (a) 2020, China (38)	30	47	16/14	54.6 ± 14.2 NR NR NR NR	NR NR NR NR	30	34	20/10	102.5 ± 30.8 NR NR NR NR	NR NR NR NR
Zhou Y et al. (b) 2020, China (38)	30	47	16/14	54.6 ± 14.2 NR NR NR NR	NR NR NR NR	32	44	14/18	79.5 ± 38.2 NR NR NR NR	NR NR NR NR
Anderson EW et al., 2021, USA (39)	74	36	NR	NR NR NR NR NR	NR 0.031 ± 0.009 NR 9.13 ± 4.44	74	38	NR	NR NR NR NR NR	NR 0.045 ± 0.02 NR 18.0 ± 10.9
Eryavuz Onmaz D et al. (a) 2021, Turkey (40)	50	42	27/23	12,215 ± 4,255 317 ± 146 6.07 ± 2.65 2.1 ± 0.81 6.34 ± 3.08	19.3 ± 6.7 0.1125 ± 0.0175 NR NR	35	40	24/11	11,694 ± 7,076 1,217 ± 918 4.39 ± 2.04 2.62 ± 1.65 5.24 ± 2.95	23.2 ± 8.7 0.281 ± 0.230 NR NR
Eryavuz Onmaz D et al. (b) 2021, Turkey (40)	50	42	27/23	12,215 ± 4,255 317 ± 146 6.07 ± 2.65 2.1 ± 0.81 6.34 ± 3.08	19.3 ± 6.7 0.1125 ± 0.0175 NR NR	50	40	31/19	9,165 ± 4,250 431 ± 163 5.18 ± 3.00 2.73 ± 1.73 5.24 ± 2.95	17.7 ± 4.9 0.0775 ± 0.05 NR NR
Kor A et al., 2022, Turkey (41)	41	54	10/31	17,175 ± 18,310 707 ± 932 NR NR NR	NR 0.0333 ± 0.0195 NR NR	50	59	13/37	10,244 ± 9,699 669 ± 761 NR NR NR	NR 0.0628 ± 0.0726 NR NR
Eryavuz Onmaz D et al., 2022, Turkey (42)	120	41	51/67	10,908 ± 4,274 280 ± 123 4.67 ± 3.11 2.25 ± 1.21 5.53 ± 3.15	17.7 ± 7.4 0.039 ± 0.02 NR NR	120	41	55/63	9,444 ± 4,334 781 ± 520 6.00 ± 3.27 4.52 ± 2.71 6.67 ± 3.81	27.1 ± 16.3 0.078 ± 0.0425 NR NR
Pellicano C et al., 2022, Italy (43)	20	59	2/18	NR NR 54.2 ± 14.2 NR NR	NR NR NR NR	52	57	5/47	NR NR 68.6 ± 24.8 NR NR	NR NR NR NR
Apaydın H et al., 2022, Turkey (44)	42	54	3/39	12,804 ± 2,421 413 ± 104 NR NR NR	NR 0.0333 ± 0.0074 NR NR	34	53	2/32	10,700 ± 2,313 488 ± 165 NR NR NR	NR 0.0433 ± 0.0222 NR NR
Jeon C et al., 2023, Republic of Korea (45)	22	33	22/0	NR 445 ± 91 NR NR NR	NR NR NR NR	87	39	87/0	NR 474 ± 147 NR NR NR	NR NR NR NR
Eryavuz Onmaz D et al., 2023, Turkey (46)	80	51	4/76	54 ± 28 1.06 ± 0.33 19.3 ± 11.4 16.6 ± 15.8 35.2 ± 34.3	90 ± 44 0.0204 ± 0.0107 18.3 ± 12 5.42 ± 3.84	80	52	3/77	38 ± 26 3.49 ± 2.97 30.3 ± 21.7 21.3 ± 19.3 47.2 ± 27.4	165 ± 61 0.0806 ± 0.0329 10.1 ± 5.9 6.59 ± 5.01
Park Y et al., 2023, Republic of Korea (47)	30	NR	NR	30.5 ± 3.8 19.8 ± 5 NR NR NR	3.29 ± 2.5 0.676 ± 0.202 NR NR	81	53	2/79	18 ± 4.2 17.4 ± 6.8 NR NR NR	10.8 ± 6.7 0.892 ± 0.919 NR NR
Tezkan D et al., 2023, Turkey (48)	80	35	38/42	10,032 ± 5,107 249 ± 156 4.46 ± 3.34 4.03 ± 1.84 5.85 ± 3.77	10.96 ± 3.82 0.026 ± 0.14 NR NR	81	34	40/41	7,673 ± 4,400 406 ± 288 2.60 ± 1.96 7.39 ± 2.84 7.17 ± 2.89	21.37 ± 15.78 0.0728 ± 0.0695 NR NR
Yurt EF et al., 2023, Turkey (49)	50	42	22/28	10,958 ± 2,253 366 ± 100 NR NR NR	NR 0.0337 ± 0.093 NR NR	104	45	50/54	9,530 ± 1,869 414 ± 86 NR NR NR	NR 0.0432 ± 0.0153 NR NR

F, female; M, male; NR, not reported; Trp, tryptophan; Kyn, kynurenine; KynA, kynurenine acid; QuinA, quinolinic acid, 3HK, 3-hydroxykynurenine; 3HAA, 3-hydroxyanthranilic acid; Kyn/Trp, kynurenine to tryptophan ratio; KynA/Kyn, kynurenine acid to kynurenine ratio; QuinA/KynA, quinolinic acid to kynurenine acid ratio.

Concentrations are expressed in a) µmol/L or ng/mL for Trp and Kyn, and b) nmol/L or ng/mL for KynA, 3HAA, 3HK, and Quin A.

### Tryptophan

We identified 21 studies (23 comparator groups) reporting tryptophan concentrations in a total of 1,526 RD patients (mean age 48 years, 24% males) and 1,404 healthy controls (mean age 43 years, 38% males; **Table 1**) (26–38, 40–42, 44, 46–49). Ten were conducted in Europe (26–29, 32–37), and 11 in Asia (30, 31, 38, 40–42, 44, 46–49). Six study groups included patients with RA (28, 31, 34, 36, 38, 41), five with pSS (29, 33, 44, 46, 47), four with SLE (27, 30, 32, 35), three with AS (38, 40), two with SSc (26, 37), and one with BD (42), FMF (48), and axSpA (49), respectively. Liquid chromatography was used in 21 study groups (27–38, 40–42, 44, 46, 48, 49), a spectrofluorimetric assay in one (26), and an enzyme-linked immunosorbent assay (ELISA) in the remaining one (47). Among the liquid chromatography-based assays, seven used fluorimetric detection (27–33), and the remaining 14 mass spectrometry (34–38, 40–42, 44, 46, 48, 49). Measurements were conducted in serum in 17 study groups (26–33, 36, 38, 40, 42, 46–48), and plasma in the remaining six (34, 35, 37, 41, 44, 49). The risk of bias (**Supplementary Table 3**) was moderate in 13 studies (26–33, 35, 37, 41, 44, 47), and low in the remaining eight (34, 36, 38, 40, 42, 46, 48, 49).

**Figure 3:**
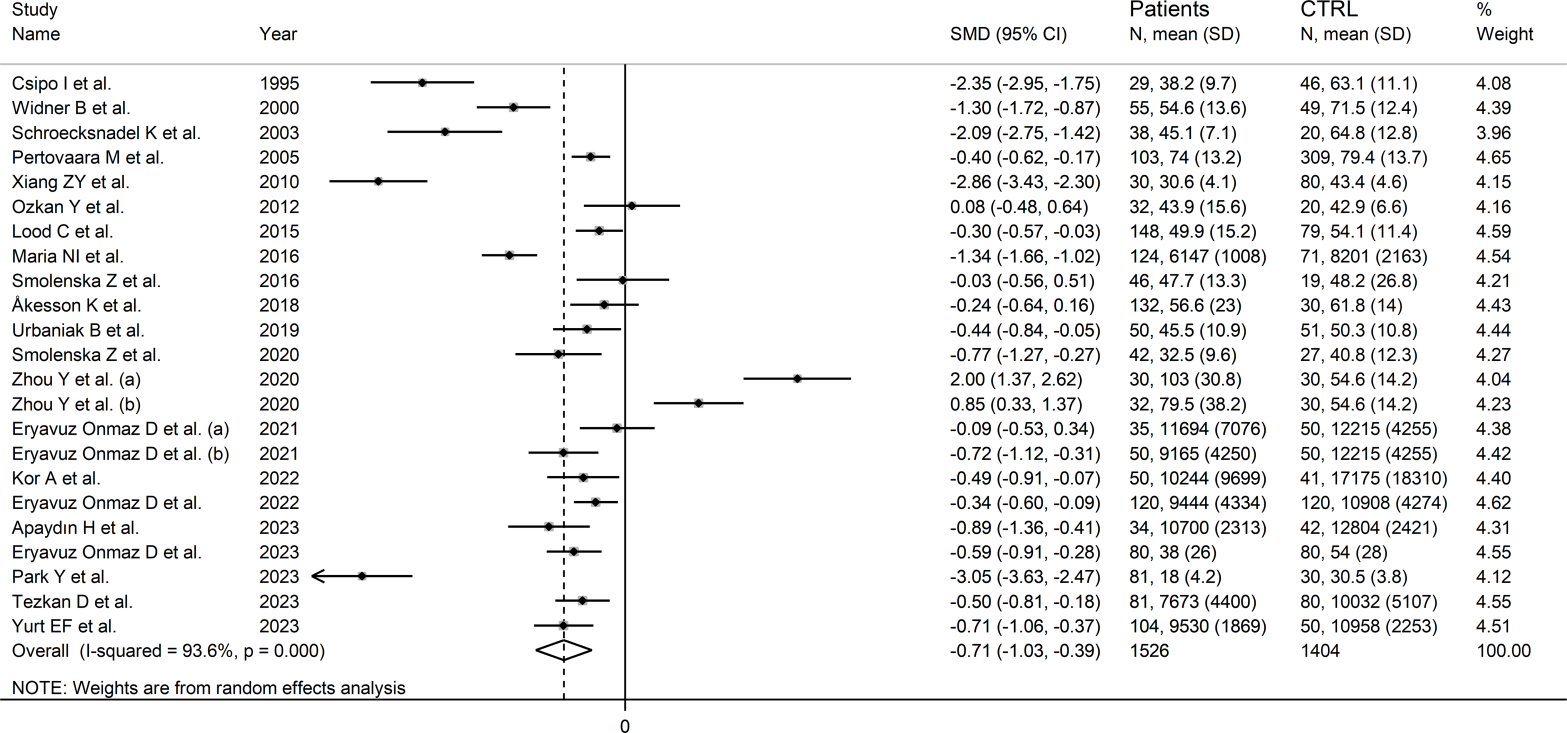
Forest plot of the standard mean differences in tryptophan concentrations between patients with rheumatic disease and healthy controls.

The forest plot showed that tryptophan concentrations were significantly lower in RD patients compared to controls (SMD=-0.71, 95% CI -1.03 to -0.39, p<0.001; I^2^ = 93.6%, p<0.001; **Figure 3**). The pooled SMD values were not influenced by individual studies (range between -0.82 and -0.61; **Supplementary Figure 1**). There was no publication bias (Begg’s test, p=0.32; Egger’s test, p=0.30). The “trim-and-fill” method did not identify any missing study to be added to the funnel plot to ensure symmetry (**Supplementary Figure 2**).

In meta-regression, there were non-significant associations between effect size and age (t=-1.60, p=0.13), publication year (t=1.89, p=0.07), sample size (t=0.04, p=0.97), RD duration (t=1.68, p=0.12), CRP (t=1.96, p=0.08), or use of DMARDs (t=0.72, p=0.49) and corticosteroids (t=-0.05, p=0.97). By contrast, the effect size was significantly associated with the (male/female RD patients)/(male/female controls) ratio (t=2.25, p=0.04).

**Figure 4:**
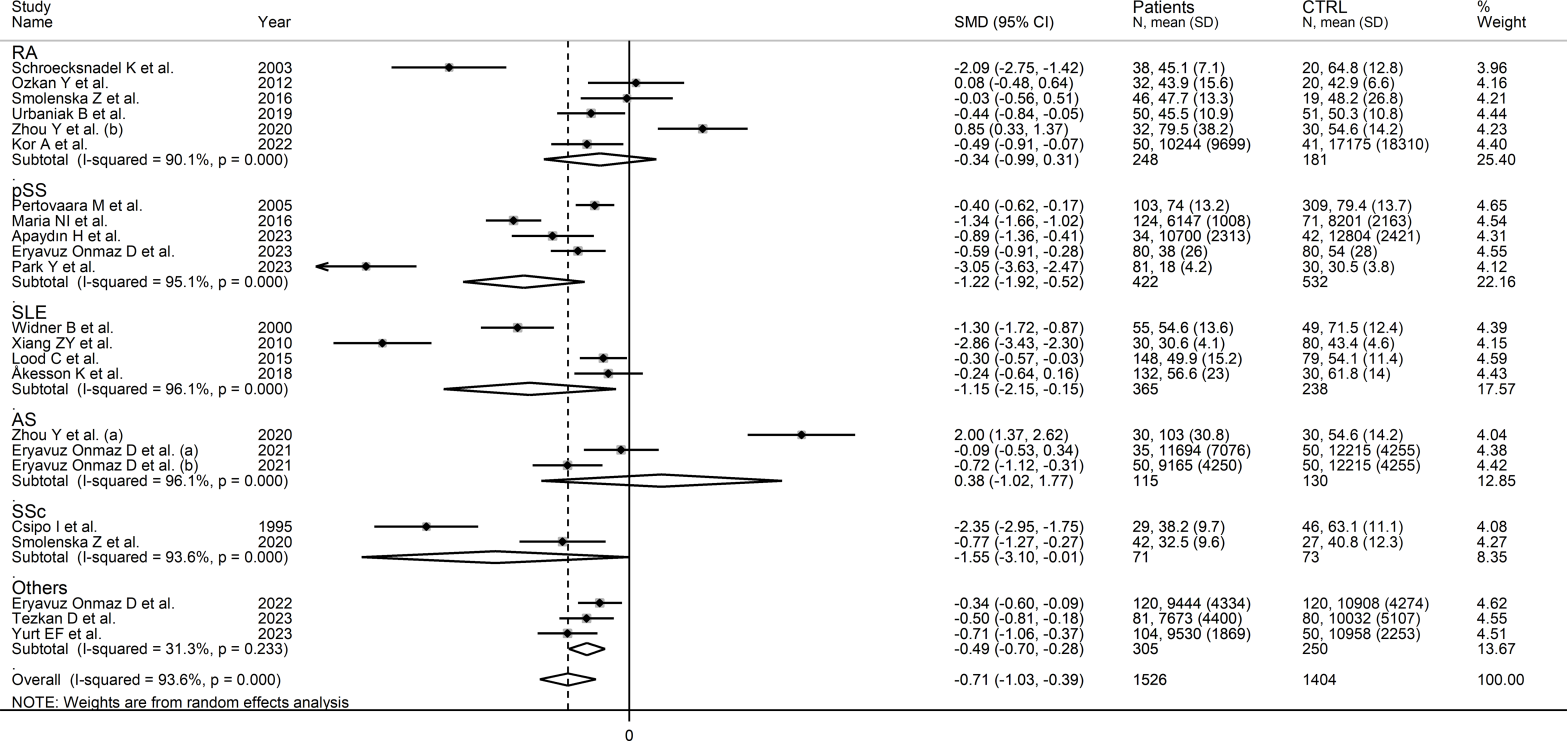
Forest plot of studies investigating tryptophan concentrations in patients and controls according to the type of rheumatic disease.

In subgroup analysis, the pooled SMD was statistically significant in studies investigating pSS (SMD=-1.22, 95% CI -1.92 to -0.52, p<0.001; I^2^ = 95.1%, p<0.001), SLE (SMD=-1.15, 95% CI -2.15 to -0.15, p=0.02; I^2^ = 96.1%, p<0.001), and SSc patients (SMD=1.55, 95% CI -3.10 to -0.01, p=0.049; I^2^ = 93.6%, p<0.001), but not RA (SMD=-0.34, 95% CI -0.99 to 0.31, p=0.31; I^2^ = 90.1%, p<0.001), or AS patients (SMD=0.38, 95% CI -1.02 to 1.77, p=0.60; I^2^ = 96.1%, p<0.001; **Figure 4**). In addition, the pooled SMD was statistically significant in studies of patients with autoimmune disease (SMD=-0.93, 95% CI -1.92 to -0.52, p<0.001; I^2^ = 93.8%, p<0.001), but not mixed autoimmune-autoinflammatory disease (SMD=-0.01, 95% CI -0.69 to 0.66, p=0.97; I^2^ = 93.6%, p<0.001; **Supplementary Figure 3**). There were non-significant differences (p=0.46) in pooled SMD between European (SMD=-0.89, 95% CI -1.29 to -0.49, p<0.001; I^2^ = 90.7%, p<0.001) and Asian studies (SMD=-0.56, 95% CI -1.07 to -0.05, p=0.03; I^2^ = 95.0%, p<0.001). By contrast, there was a significant difference (p=0.006) between the pooled SMD of studies using liquid chromatography (SMD=-0.53, 95% CI -0.82 to -0.24, p<0.001; I^2^ = 91.6%, p<0.001) and other assays (SMD=-2.71, 95% CI -3.39 to -2.02, p<0.001; I^2^ = 62.6%, p=0.10) with a relatively lower heterogeneity in the latter subgroup. In addition, in studies using liquid chromatography the pooled SMD was statistically significant in those using fluorimetric detection (SMD=-1.15, 95% CI -1.77 to -0.53, p<0.001; I^2^ = 94.8%, p<0.001) but not in those using mass spectrometry detection (SMD=-0.24, 95% CI -0.54 to 0.05, p=0.10; I^2^ = 86.6%, p<0.001), and the difference between the effect sizes was also statistically significant (p=0.03). Finally, there were non-significant differences (p=0.63) in pooled SMD between studies investigating plasma (SMD=-0.53, 95% CI -0.78 to -0.28, p<0.001; I^2^ = 48.1%, p=0.09) and those investigating serum (SMD=-0.78, 95% CI -1.20 to -0.36, p<0.001; I^2^ = 93.6%, p<0.001), with a lower between-study variance in the former subgroup.

The certainty of evidence remained low (rating 2, ⊕⊕⊝⊝) after considering the low-moderate risk of bias in all studies (no rating change), the high but partly explainable heterogeneity (no rating change), the lack of indirectness (no rating change), the relatively low imprecision (confidence intervals not crossing the threshold, no rating change), the moderate effect size (SMD=-0.71, no rating change) (50), and the absence of publication bias (no rating change).

### Kynurenine

Seventeen studies (18 comparator groups) investigated kynurenine concentrations in a total of 1,384 RD patients (mean age 48 years, 29% males) and 1,223 healthy controls (mean age 44 years, 40% males; **Table 1**) (27–33, 35, 40–42, 44–49). Eleven studies were conducted in Asia (30, 31, 40–42, 44–49), and six in Europe (27–29, 32, 33, 35). Five study groups included patients with pSS (29, 33, 44, 46, 47), four with SLE (27, 30, 32, 35), three with AS (40, 45), three with RA (28, 31, 41), and one with BD (42), FMF (48), and axSpA (49), respectively. Liquid chromatography was used in 15 study groups (27–33, 40–42, 44, 46, 48, 49), ELISA in two (45, 47), and gas chromatography in one (35). In liquid chromatography studies, seven used mass spectrometry (40–42, 44, 46, 48, 49), six ultraviolet (27–29, 31–33), and one fluorimetric detection (30). Kynurenine was measured in serum in 14 study groups (27–33, 40, 42, 45–48), and plasma in the remaining four (35, 41, 44, 49). The risk of bias (**Supplementary Table 3**) was moderate in 12 studies (27–33, 35, 41, 44, 45, 47), and low in the remaining five (40, 42, 46, 48, 49).

**Figure 5:**
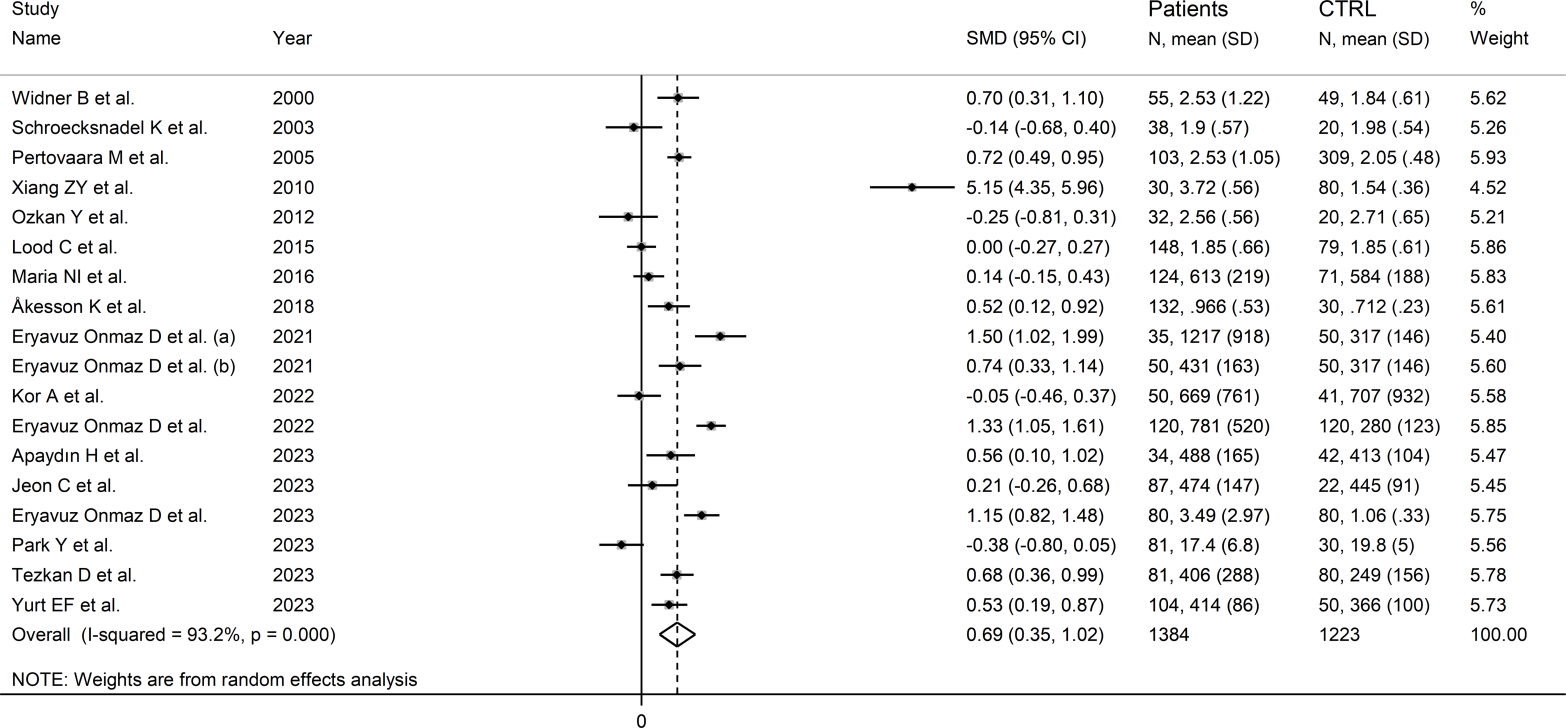
Forest plot of the standard mean differences in kynurenine concentrations between patients with rheumatic disease and healthy controls.

The forest plot showed that kynurenine concentrations were significantly higher in RD patients compared to healthy controls (SMD=0.69, 95% CI 0.35 to 1.02, p<0.001; I^2^ = 93.2%, p<0.001; **Figure 5**). Sensitivity analysis showed stability of the results as the effect size ranged between 0.48 and 0.75 (**Supplementary Figure 4**). There was no publication bias (Begg’s test, p=0.88; Egger’s test, p=0.46). Although the “trim-and-fill” method failed to identify missing studies to be added to the funnel plot to ensure symmetry (**Supplementary Figure 5**), it highlighted the distortive effects of the study of Xiang et al. (30). The removal of this study from the analysis attenuated the effect size (SMD=0.48, 95% CI 0.23 to 0.73, p=0.001; I^2^ = 87.3%, p<0.001).

In meta-regression, there were non-significant associations between effect size and age (t=-0.53, p=0.61), sex (t=-0.95, p=0.36), publication year (t=-0.44, p=0.67), sample size (t=0.11, p=0.91), CRP (t=-1.68, p=0.14), RD duration (t=0.23, p=0.82), or use of DMARDs (t=-1.17, p=0.27) and corticosteroids (t=0.23, p=0.82).

**Figure 6:**
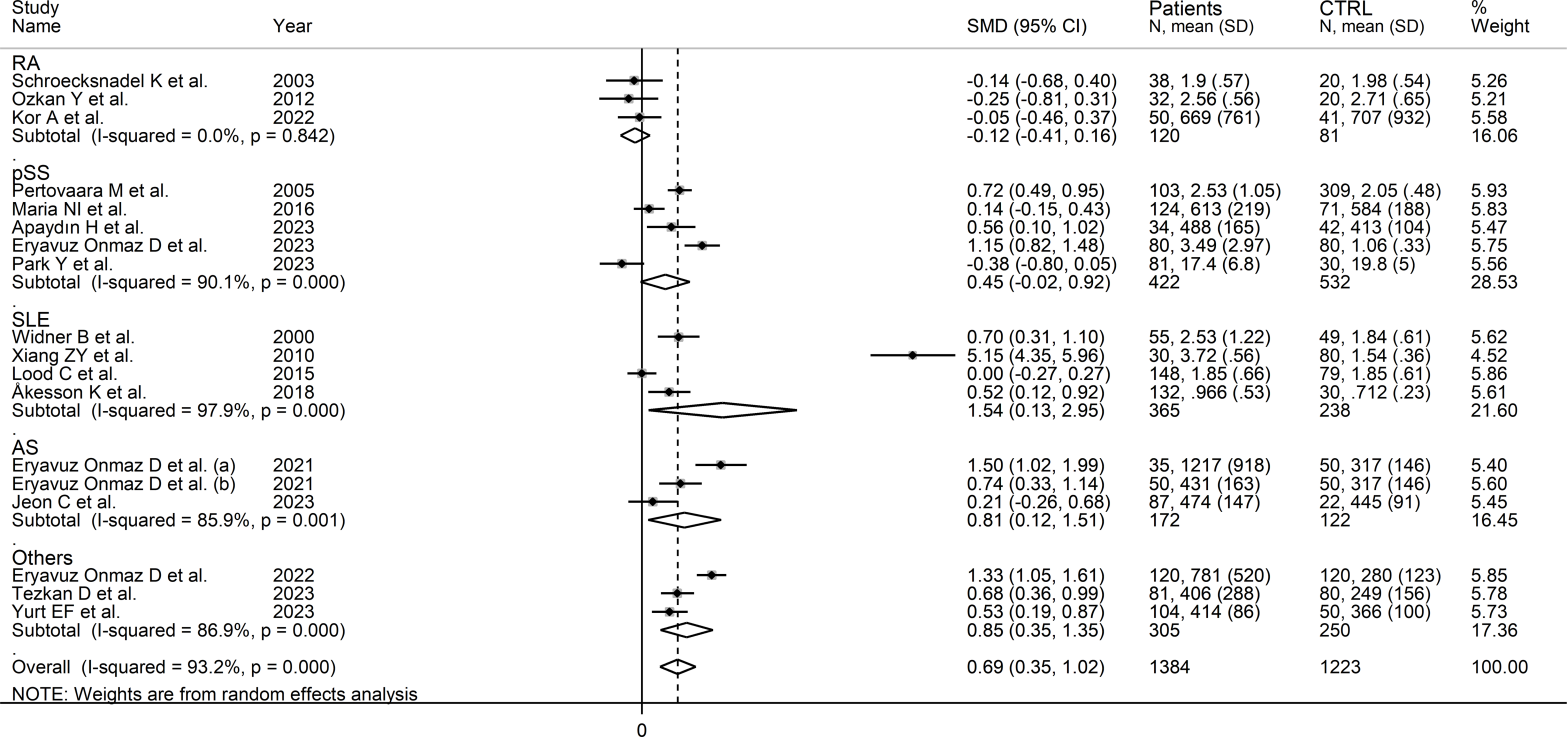
Forest plot of studies investigating kynurenine concentrations in patients and controls according to type of rheumatic disease.

In subgroup analysis, the pooled SMD was statistically significant in studies of SLE (SMD=1.54, 95% CI 0.13 to 2.95, p=0.03; I^2^ = 97.9%, p<0.001), and AS (SMD=0.81, 95% CI 0.12 to 1.51, p=0.02; I^2^ = 85.9%, p<0.001), but not RA (SMD=-0.12, 95% CI -0.41 to 0.16, p=0.39; I^2^ = 0.0%, p=0.84), or pSS (SMD=0.45, 95% CI -0.02 to 0.92, p=0.06; I^2^ = 90.1%, p<0.001; **Figure 6**), with a virtually absent heterogeneity in the RA subgroup. There were non-significant differences (p=0.82) in pooled SMD between studies in patients with autoimmune disease (SMD=0.62, 95% CI 0.16 to 1.09, p=0.009; I^2^ = 94.5%, p<0.001) and mixed autoimmune-autoinflammatory disease (SMD=0.86, 95% CI 0.41 to 1.32, p<0.001; I^2^ = 85.8%, p<0.001; **Supplementary Figure 6**). Furthermore, there were non-significant differences (p=0.34) in pooled SMD between Asian (SMD=0.89, 95% CI 0.38 to 1.40, p=0.001; I^2^ = 94.7%, p<0.001) and European studies (SMD=0.34, 95% CI 0.04 to 0.64, p=0.03; I^2^ = 80.0%, p<0.001). By contrast, the pooled SMD was statistically significant in studies using liquid chromatography (SMD=0.80, 95% CI 0.42 to 1.18, p<0.001; I^2^ = 93.8%, p<0.001) but not in those using other methods (SMD=0.12, 95% CI -0.42 to 0.65, p=0.66; I^2^ = 78.4%, p=0.010). In studies using liquid chromatography, the pooled SMD was significantly different with mass spectrometry (SMD=0.81, 95% CI 0.47 to 1.14, p<0.001; I^2^ = 84.6%, p<0.001) but not ultraviolet detection (SMD=0.23, 95% CI -0.11 to 0.56, p=0.19; I^2^ = 82.6%, p<0.001), and the difference between the effect sizes was statistically significant (p=0.038). Finally, there were non-significant differences (p=0.56) in pooled SMD between studies in plasma (SMD=0.39, 95% CI 0.11 to 0.67, p=0.006; I^2^ = 48.2%, p=0.12) and serum (SMD=0.78, 95% CI 0.36 to 1.20, p<0.001; I^2^ = 94.6%, p<0.001), with a lower between-study variance in the plasma subgroup.

The certainty of evidence remained low (rating 2, ⊕⊕⊝⊝) after considering the low-moderate risk of bias in all studies (no rating change), the high but partly explainable heterogeneity (no rating change), the lack of indirectness (no rating change), the relatively low imprecision (confidence intervals not crossing the threshold, no rating change), the moderate effect size (SMD=0.69, no rating change) (50), and the absence of publication bias (no rating change).

### Kynurenine to tryptophan ratio

Seventeen studies (18 comparator groups) investigated the kynurenine to tryptophan ratio in a total of 1,371 RD patients (mean age 48 years, 24% males; **Table 1**) and 1,275 healthy controls (mean age 44 years, 39% males) (27–33, 35, 39–42, 44, 46–49). Ten studies were conducted in Asia (30, 31, 40–42, 44, 46–49), six in Europe (27–29, 32, 33, 35), and one in America (39). Five study groups included participants with pSS (29, 33, 44, 46, 47), five with SLE (27, 30, 32, 35, 39), three with RA (28, 31, 41), two with AS (40), and one with BD (42), FMF (48), and axSpA (49), respectively. Liquid chromatography was used in 16 study groups (27–33, 39–42, 44, 46, 48, 49), whereas ELISA (47) and gas chromatography (35) were used in the remaining two. Among the studies using liquid chromatography, the detection method included mass spectrometry in nine (39–42, 44, 46, 48, 49), ultraviolet in four (27–29, 32), and fluorimetric detection in the remaining three (30, 31, 33). Serum was the biological matrix in 14 study groups (27–33, 39, 40, 42, 46–48), and plasma in the remaining four (35, 41, 44, 49). The risk of bias was moderate in 11 studies (27–33, 35, 41, 44, 47) and low in the remaining six (39, 40, 42, 46, 48, 49) (**Supplementary Table 3**).

**Figure 7:**
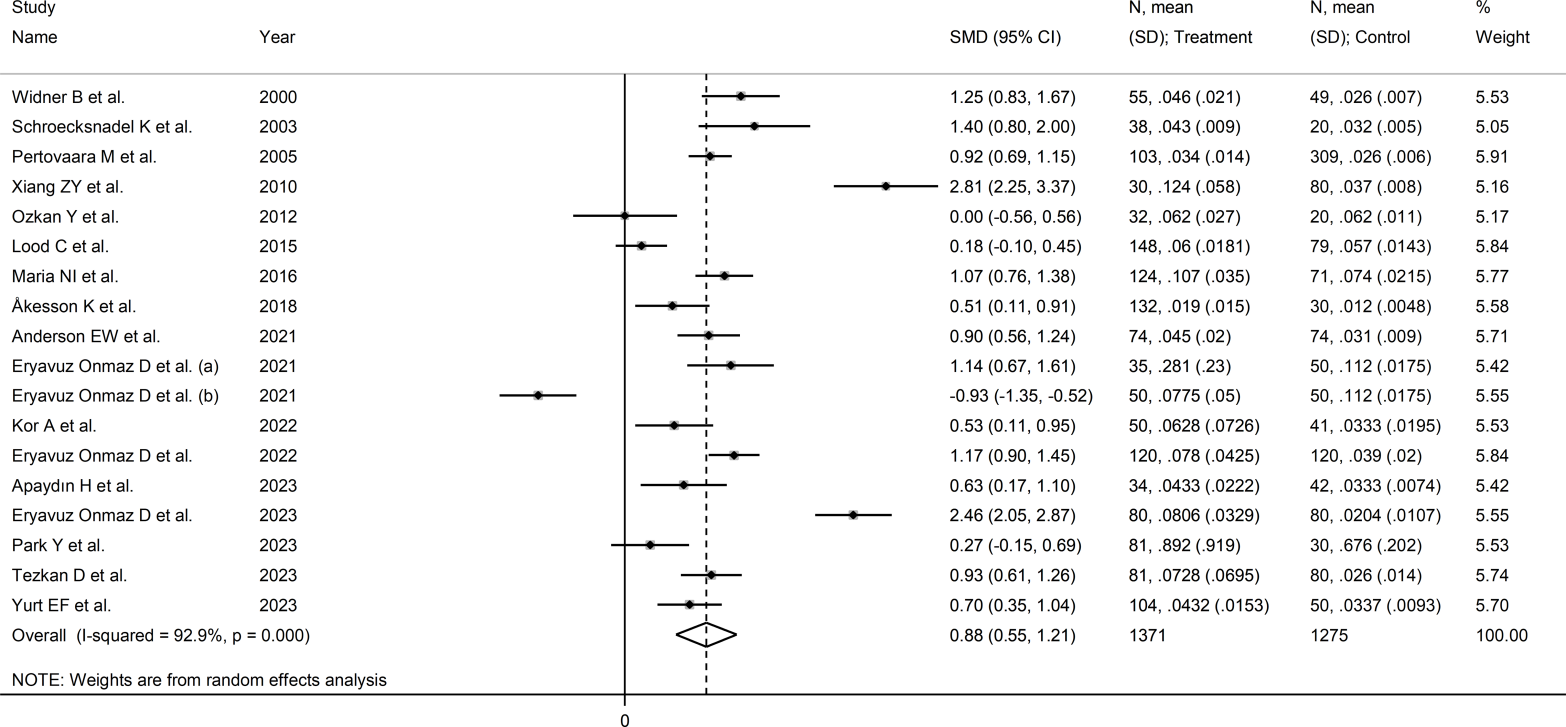
Forest plot of studies reporting the kynurenine/tryptophan ratio in patients with rheumatic disease and healthy controls.

The forest plot showed that the kynurenine to tryptophan ratio was significantly higher in RD patients compared to controls (SMD=0.88, 95% CI 0.55 to 1.21, p<0.001; I^2^ = 92.9%, p<0.001; **Figure 7**). The effect size ranged between 0.77 and 0.98 in sensitivity analysis, highlighting the stability of the results (**Supplementary Figure 7**). There was no publication bias (Begg’s test, p=1.0; Egger’s test, p=0.68). Accordingly, the “trim-and-fill” method did not identify any missing study to be added to the funnel plot to ensure symmetry (**Supplementary Figure 8**).

No significant associations were observed in meta-regression between effect size and age (t=0.83, p=0.42), sex (t=-1.62, p=0.13), publication year (t=-0.91, p=0.37), sample size (t=0.28, p=0.78), RD duration (t=-0.10, p=0.93), CRP (t=-1.49, p=0.18), or use of DMARDs (t=0.70, p=0.51) and corticosteroids (t=0.19, p=0.85).

**Figure 8:**
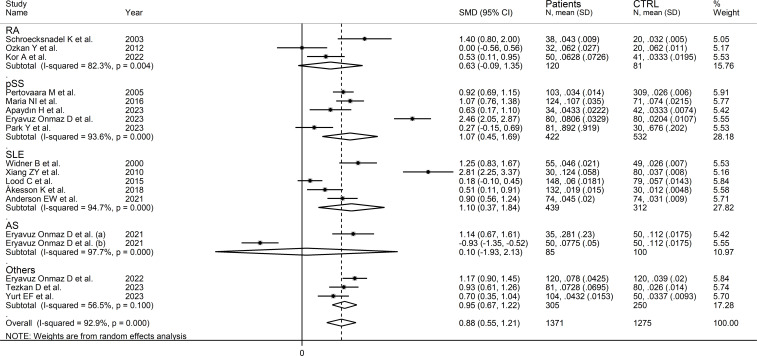
Forest plot of studies reporting the kynurenine/tryptophan ratio in patients and controls according to the type of rheumatic disease.

In subgroup analysis, the pooled SMD was statistically significant in studies of SLE (SMD=1.10, 95% CI 0.37 to 1.84, p=0.003; I^2^ = 94.7%, p<0.001) and pSS (SMD=1.07, 95% CI 0.45 to 1.69, p=0.001; I^2^ = 93.6%, p<0.001), but not RA (SMD=-0.63, 95% CI -0.09 to 1.35, p=0.08; I^2^ = 82.3%, p<0.001) or AS (SMD=0.10, 95% CI -0.93 to 2.13, p=0.92; I^2^ = 97.7%, p<0.001, **Figure 8**). In addition, the pooled SMD was statistically significant in studies of patients with autoimmune disease (SMD=0.98, 95% CI 0.60 to 1.37, p<0.001; I^2^ = 92.5%, p<0.001) but not of patients with autoimmune-autoinflammatory diseases (SMD=0.52, 95% CI -0.39 to 1.43, p=0.26; I^2^ = 96.0%, p<0.001; **Supplementary Figure 9**). There were non-significant differences (p=0.99) in pooled SMD between European (SMD=0.86, 95% CI 0.50 to 1.22, p<0.001; I^2^ = 85.3%, p<0.001) and Asian studies (SMD=0.88, 95% CI 0.33 to 1.43, p=0.002; I^2^ = 95.1%, p<0.001). Similarly, the pooled SMD was non-significantly different (0.40) between studies using liquid chromatography (SMD=0.94, 95% CI 0.58 to 1.30, p<0.001; I^2^ = 93.4%, p<0.001) and other methods (SMD=0.40, 95% CI 0.11 to 0.69, p=0.007; I^2^ = 0.0%, p=0.42), with a virtually absent heterogeneity in the latter subgroup. In liquid chromatography studies, the pooled SMD was statistically significant in studies using mass spectrometry (SMD=0.84, 95% CI 0.32 to 1.36, p=0.002; I^2^ = 94.3%, p<0.001) and ultraviolet detection (SMD=0.90, 95% CI 0.37 to 1.43, p=0.001; I^2^ = 89.5%, p<0.001), but not in those using fluorimetric detection (SMD=1.29, 95% CI -0.06 to 2.64, p=0.06; I^2^ = 96.0%, p<0.001). Finally, there were non-significant differences (p=0.45) in pooled SMD between studies analysing plasma (SMD=0.96, 95% CI 0.55 to 1.37, p<0.001; I^2^ = 94.4%, p<0.001) and those on serum (SMD=0.60, 95% CI 0.40 to 0.80, p<0.001; I^2^ = 0.0%, p=0.90), with a virtually absent between study-variance in the serum subgroup.

The certainty of evidence was upgraded to moderate (rating 3, ⊕⊕⊕⊝) after considering the low-moderate risk of bias in all studies (no rating change), the high but partly explainable heterogeneity (no rating change), the lack of indirectness (no rating change), the relatively low imprecision (confidence intervals not crossing the threshold, no rating change), the large effect size (SMD=0.88, upgrade one level) (50), and the absence of publication bias (no rating change).

### Kynurenic acid

Six studies (seven comparator groups) reported kynurenic acid concentrations in a total of 448 RD patients (mean age 43 years, 37% males) and 480 healthy controls (mean age 41 years, 39% males; **Table 1**) (30, 40, 42, 43, 46, 48). Five studies were conducted in Asia (30, 40, 42, 46, 48), and the remaining one in Europe (43). Two study groups included individuals with AS (40), and one with SLE (30), SSc (43), pSS (46), BD (42), and FMF (48), respectively. Liquid chromatography was used in six study groups (30, 40, 42, 46, 48), and ELISA in the remaining one (43). In liquid chromatography studies, five study groups using mass spectrometry for detection (40, 42, 46, 48), and the remaining one fluorimetry (30). All studies investigated serum. The risk of bias (**Supplementary Table 3**)was moderate in one study (30), and low in the remaining five (40, 42, 43, 46, 48).

**Figure 9:**
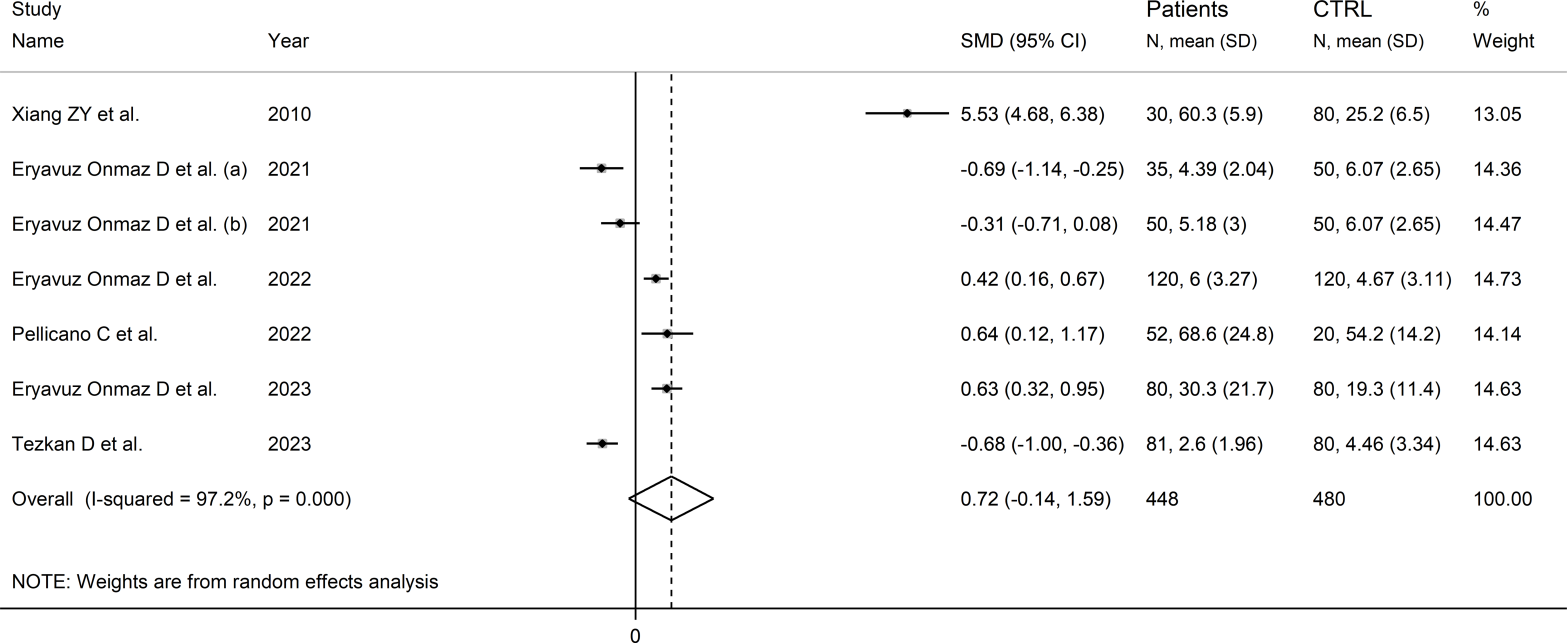
Forest plot of studies reporting kynurenic acid concentrations in patients with rheumatic disease and healthy controls.

The forest plot showed that kynurenic acid concentrations were not significantly different between RD patients and controls (SMD=0.72, 95% CI -0.14 to 1.59, p=0.10; I^2^ = 96.7%, p<0.001; **Figure 9**). Sensitivity analysis showed that the study of Xian et al. had a significant effect on the corresponding pooled SMD direction (30) (**Supplementary Figure 10**). Its removal reduced the effect size (SMD=0.00, 95% CI -0.50 to 0.50, p=1.00).

The limited number of studies prevented the assessment of publication bias and the conduct of meta-regression and subgroup analyses.

The certainty of evidence was downgraded to extremely low (rating 0, ⊝⊝⊝⊝) after considering the low-moderate risk of bias in all studies (no rating change), the high and unexplainable heterogeneity (downgrade one level), the lack of indirectness (no rating change), and the lack of assessment of publication bias (downgrade one level).

### 3-hydroxyanthranilic acid

Four studies (five comparator groups), all conducted in Asia, reported 3-hydroxyanthranilic acid concentrations in a total of 366 patients with RD (mean age 42 years, 51% males) and 380 healthy controls (mean age 42 years, 39% males; **Table 1**) (40, 42, 46, 48). Two study groups included individuals with AS (40), and one with pSS (46), BD (42), and FMF (48), respectively. In all studies, measurements were conducted in serum using liquid chromatography with mass spectrometry detection. The risk of bias (**Supplementary Figure 3**) was low in all studies (40, 42, 46, 48).

**Figure 10:**
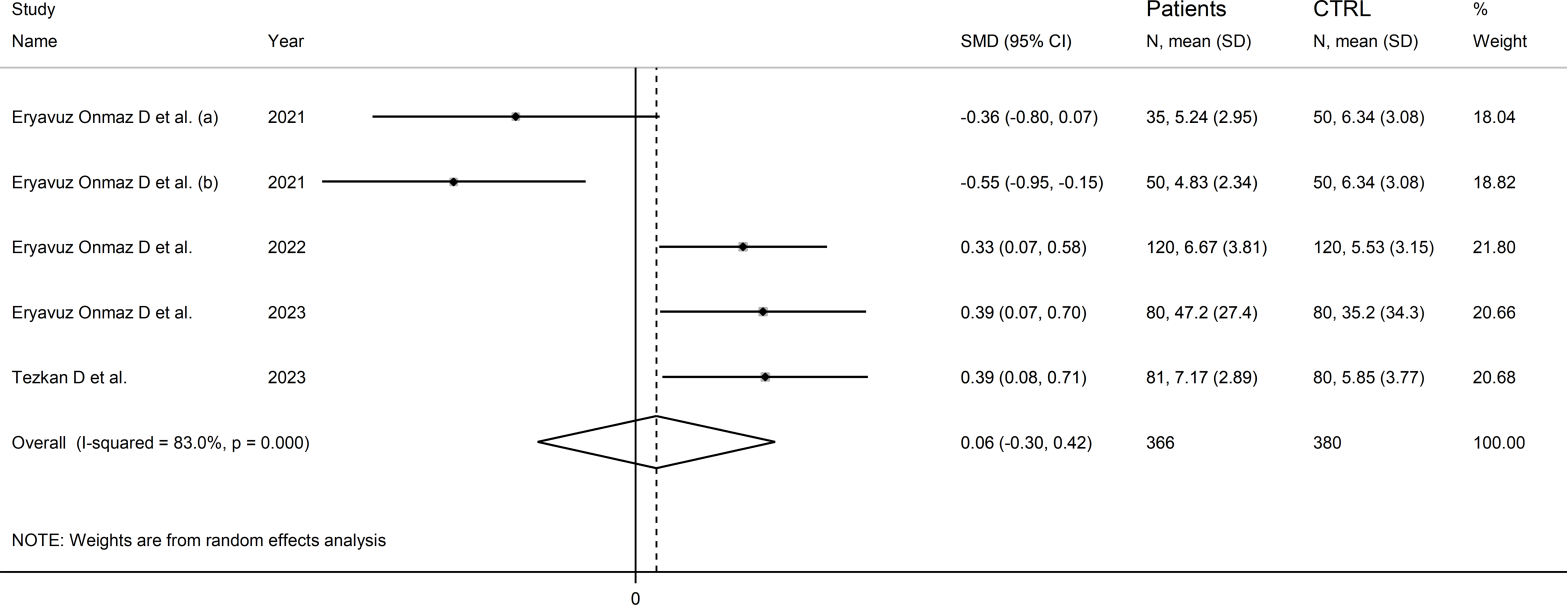
Forest plot of studies reporting 3-hydroxyanthranilic acid concentrations in patients with rheumatic disease and healthy controls.

The forest plot showed the absence of significant between-group differences in 3-hydroxyanthranilic acid concentrations (SMD=0.06, 95% CI -0.30 to 0.42, p=0.73; I^2^ = 83.0%, p<0.001; **Figure 10**). The corresponding SMD values were stable in sensitivity analysis (range between -0.03 and 0.22; **Supplementary Figure 11**).

The limited number of studies prevented the assessment of publication bias and the conduct of meta-regression and subgroup analyses.

The certainty of evidence was downgraded to extremely low (rating 0, ⊝⊝⊝⊝) after considering the low-moderate risk of bias in all studies (no rating change), the high and unexplainable heterogeneity (downgrade one level), the lack of indirectness (no rating change), and the lack of assessment of publication bias (downgrade one level).

### 3-hydroxykynurenine

Four studies (five comparator groups) reported 3-hydroxykynurenine concentrations in a total of 366 RD patients (mean age 42 years, 51% males) and 380 healthy controls (mean age 42 years, 39% males; **Table 1**) (40, 42, 46, 48). Two study groups included individuals with AS (40), and one with pSS (46), BD (42), and FMF (48), respectively. In all studies, measurements were conducted in serum using liquid chromatography with mass spectrometry detection. The risk of bias (**Supplementary Table 3**) was low in all studies (40, 42, 46, 48).

**Figure 11:**
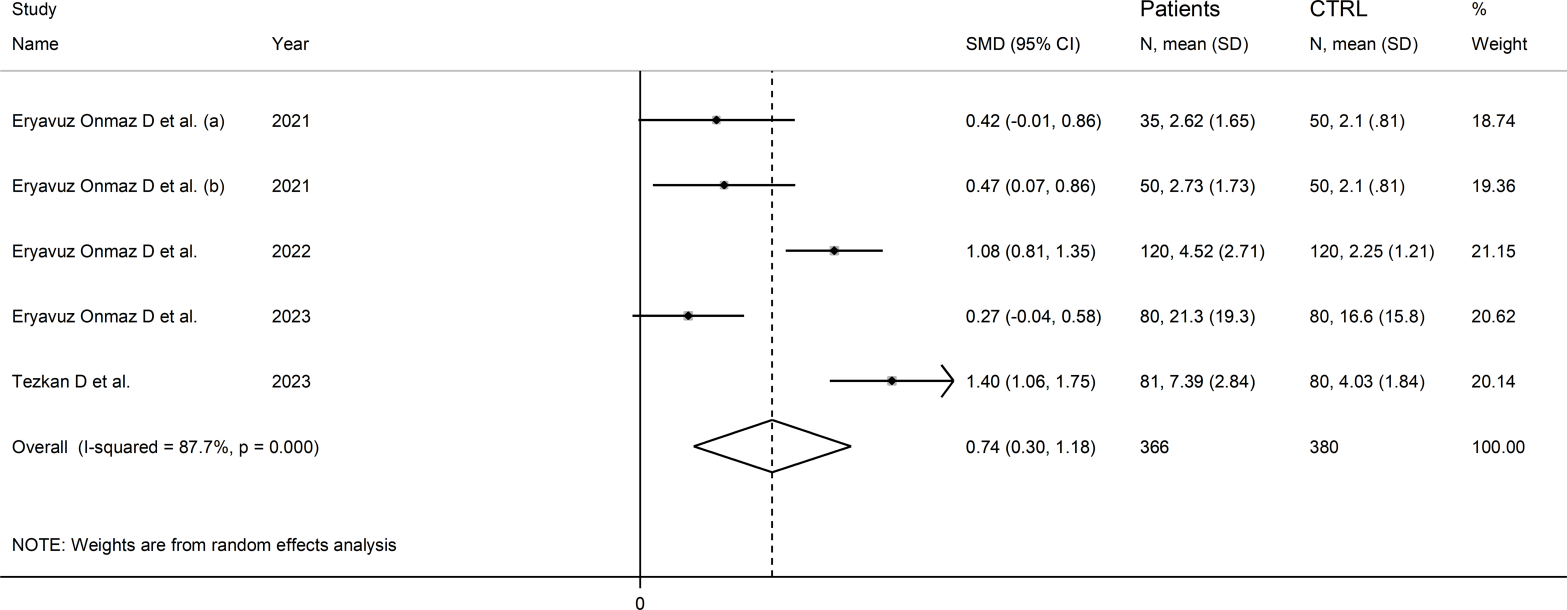
Forest plot of studies reporting 3-hydroxykynurenine concentrations in patients with rheumatic disease and healthy controls.

The forest plot showed that RD patients had significantly higher 3-hydroxykynurenine concentrations compared to healthy controls (SMD=0.74, 95% CI 0.30 to 1.18, p=0.001; I^2^ = 87.7%, p<0.001; **Figure 11**). The effect size was stable in sensitivity analysis, with a range between 0.57 and 0.86 (**Supplementary Figure 12**).

The limited number of studies prevented the assessment of publication bias and the conduct of meta-regression and subgroup analyses.

The certainty of evidence was downgraded to extremely low (rating 0, ⊝⊝⊝⊝) after considering the low-moderate risk of bias in all studies (no rating change), the high and unexplainable heterogeneity (downgrade one level), the lack of indirectness (no rating change), and the lack of assessment of publication bias (downgrade one level).

### Quinolinic acid

Six studies (seven comparator groups) reported the concentrations of quinolinic acid in a total of 579 RD patients (mean age 45 years, 35% males) and 440 controls (mean age 42 years, 39% males; **Table 1**) (35, 40, 42, 46–48). Five studies were conducted in Asia (40, 42, 46–48), and the remaining one in Europe (35). Two study groups included individuals with AS (40), two with pSS (46, 47), and one with SLE (35), one BD (42), and FMF (48), respectively. The method used was liquid chromatography with mass spectrometry in five study groups (40, 42, 46, 48), gas chromatography with mass spectrometry in one (35), and ELISA in the remaining one (47). All studies assessed serum, barring one which assessed plasma (35). The risk of bias (**Supplementary Table 3**) was moderate in two studies (35, 47), and low in the remaining four (40, 42, 46, 48).

**Figure 12:**
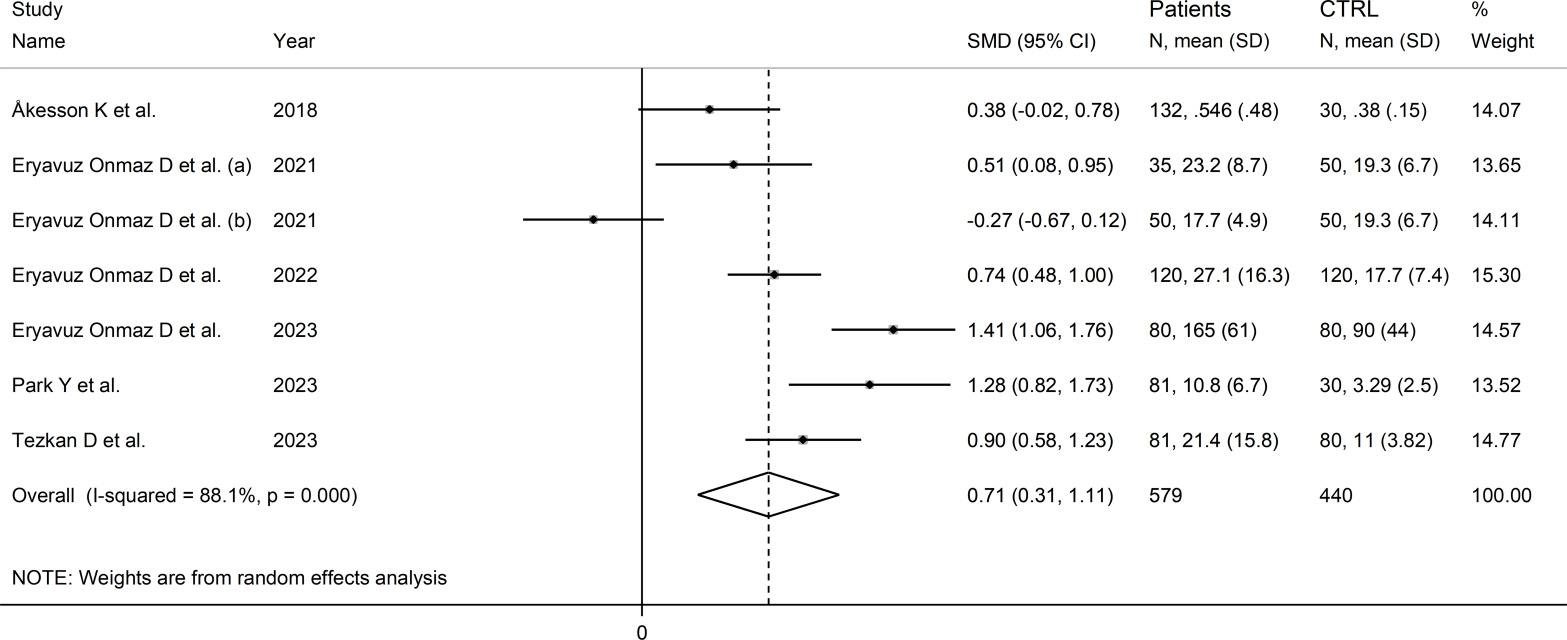
Forest plot of studies reporting the concentrations of quinolinic acid in patients with rheumatic disease and healthy controls.

The forest plot showed that the concentrations of quinolinic acid were significantly higher in RD patients compared to healthy controls (SMD=0.71, 95% CI 0.31 to 1.11, p<0.001; I^2^ = 88.1%, p<0.001; **Figure 12**). Sensitivity analysis showed that the corresponding pooled SMD values were not influenced by sequentially removing individual studies (effect size range between 0.59 and 0.87; **Supplementary Figure 13**).

The limited number of studies prevented the assessment of publication bias and the conduct of meta-regression and subgroup analyses.

The certainty of evidence was downgraded to extremely low (rating 0, ⊝⊝⊝⊝) after considering the low-moderate risk of bias in all studies (no rating change), the high and unexplainable heterogeneity (downgrade one level), the lack of indirectness (no rating change), and the lack of assessment of publication bias (downgrade one level).

### Kynurenic acid to kynurenine ratio

Two Asian studies reported the kynurenine acid to kynurenine ratio in a total of 110 RD patients (mean age 43 years, 7% males) and 160 healthy controls (mean age 47 years, 26% males; **Table 1**) (30, 46). One study included individuals with SLE (30), and one with pSS (46). Liquid chromatography was used in both studies, one with mass spectrometry detection (46), and the other with fluorimetric detection (30). Serum was analysed in both studies. The risk of bias (**Supplementary Table 3**) was moderate in one study (30) and low in the other (46).

**Figure 13:**
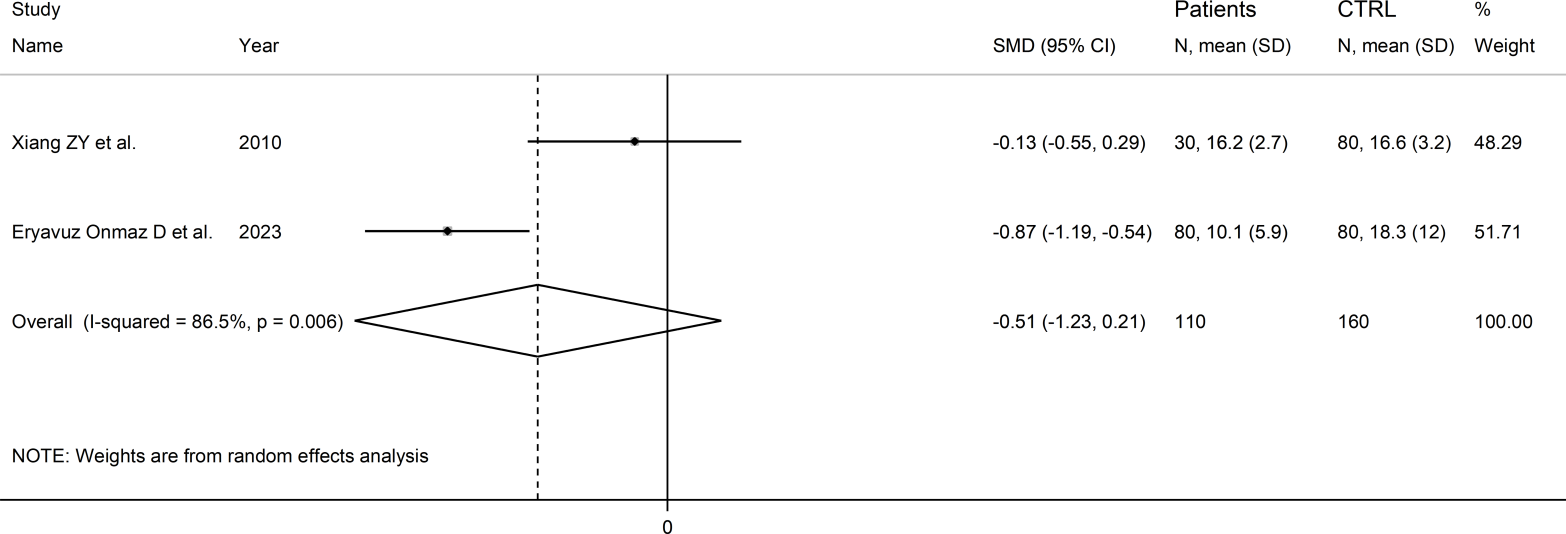
Forest plot of studies reporting the kynurenine acid to kynurenine ratio in patients with rheumatic disease and healthy controls.

The forest plot showed that the kynurenine acid to kynurenine ratio was non-significantly different between RD patients and controls (SMD=-0.51, 95% CI -1.23 to 0.21, p=0.17; I^2^ = 86.5%, p=0.006; **Figure 13**).

The limited number of studies prevented sensitivity analysis, the assessment of publication bias, and the conduct of meta-regression and subgroup analyses.

The certainty of evidence was downgraded to extremely low (rating 0, ⊝⊝⊝⊝) after considering the low-moderate risk of bias in all studies (no rating change), the high and unexplainable heterogeneity (downgrade one level), the lack of indirectness (no rating change), and the lack of assessment of publication bias (downgrade one level).

### Quinolinic acid to kynurenine acid ratio

Two studies reported the quinolinic acid to kynurenine acid ratio in a total of 154 RD patients (mean age 45 years) and 154 healthy controls (mean age 44 years; **Table 1**) (39, 46). One study was conducted in Asia (46), and the other in America (39). One study included individuals with SLE (39), and the other with pSS (46). In both studies, serum was assessed using liquid chromatography with mass spectrometry detection and the risk of bias was low (**Supplementary Figure 3**) (39, 46).

**Figure 14:**
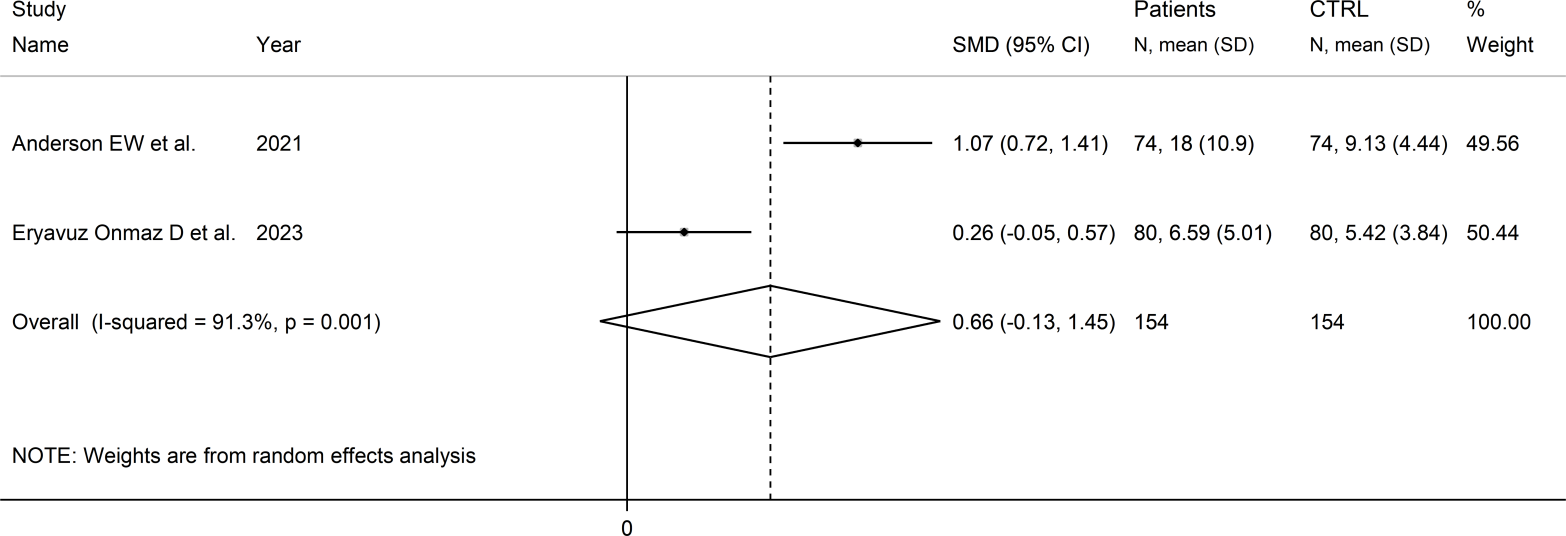
Forest plot of studies reporting the quinolinic acid to kynurenine acid ratio in patients with rheumatic disease and healthy controls.

The forest plot showed the absence of significant between-group differences in the quinolinic acid to kynurenine acid ratio (SMD=0.66, 95% CI -0.13 to 1.45, p=0.10, I^2^ = 91.3%, p=0.001; **Figure 14**).

The limited number of studies prevented sensitivity analysis, the assessment of publication, and the conduct of meta-regression and subgroup analyses.

The certainty of evidence was downgraded to extremely low (rating 0, ⊝⊝⊝⊝) after considering the low-moderate risk of bias in all studies (no rating change), the high and unexplainable heterogeneity (downgrade one level), the lack of indirectness (no rating change), and the lack of assessment of publication bias (downgrade one level).

## Discussion

In this systematic review and meta-analysis, we have observed significant alterations in metabolites within the kynurenine pathway of tryptophan metabolism in patients with RD. Specifically, RD was associated with significantly lower tryptophan concentrations and higher kynurenine, kynurenine to tryptophan ratios, 3-hydroxykynurenine, and quinolinic acid concentrations. By contrast, there were non-significant between-group differences in kynurenic acid, 3-hydroxyanthranilic acid, kynurenic acid to kynurenine ratio, or quinolinic acid to kynurenine acid ratio. In meta-regression, the SMD of tryptophan, kynurenine, and kynurenine to tryptophan ratio were not associated with age, publication year, sample size, RD duration, C-reactive protein, or use of anti-rheumatic drugs and corticosteroids. In subgroup analysis, the SMD of tryptophan, kynurenine, and kynurenine to tryptophan ratio were statistically significant in different types of RD, barring rheumatoid arthritis. Whilst our meta-analysis on tryptophan, kynurenine, and kynurenine to tryptophan ratio included a substantial number of studies, between 17 and 21, caution is required with data interpretation in view of the relatively low certainty of evidence in our analysis, particularly when investigating other metabolites, suggesting the need for additional research to confirm or refute our findings.

The biotransformation of tryptophan to kynurenine via the intermediate N′-formylkynurenine, a critical step within the kynurenine pathway, is regulated by the enzymes tryptophan 2,3-dioixygenase (TDO) in the liver and indoleamine 2,3-dioxygenase (IDO) extra-hepatically (**Figure 1**) (2, 3). A substantial number of studies have demonstrated a distinct regulation of these enzymes. TDO is upregulated by the substrate tryptophan, the cofactor heme, and glucocorticoids, and downregulated by the end-product NAD(P)H (2, 3, 51). By contrast, IDO can be inhibited by excess tryptophan, the endogenous messenger, nitric oxide, and anti-inflammatory cytokines, e.g., interleukin-4, interleukin-10, and transforming growth factor beta, and upregulated either directly or indirectly by pro-inflammatory cytokines such as interferon gamma, interferon alpha, interleukin-1β, tumour necrosis factor alpha, and interleukin-2 (2, 3, 51–53). Therefore, the observation of a relative deficiency of tryptophan and excess of kynurenine, with a consequent increase in the kynurenine to tryptophan ratio, suggests the increased contribution of extra-hepatic IDO in tryptophan metabolism in RD, possibly mediated by IDO upregulation by pro-inflammatory cytokines, a common finding in these patients (54–56). However, the putative activation of extra-hepatic IDO could also be secondary to a relative deficiency in systemic nitric oxide, a key endogenous regulator of several biological processes such as vascular homeostasis, immune function, and neuroplasticity (57, 58). This mechanism could be relevant given the evidence of a dysregulation in nitric oxide pathways in several types of RD, and the consequent increased risk of endothelial dysfunction, atherosclerosis, and cardiovascular disease in these patients (59–66). However, it is important to emphasise that the lack of information regarding the measurement of specific cytokines and nitric oxide metabolites in the studies selected in our literature search prevented the conduct of further analyses to investigate possible associations between these molecules and the observed between-group differences in tryptophan, kynurenine, and kynurenine to tryptophan ratio. Therefore, further research is warranted to investigate this important issue.

Interestingly, in meta-regression the SMD values of tryptophan, kynurenine, and kynurenine to tryptophan ratios were not significantly associated with CRP, a conventional biomarker of systemic inflammation that is routinely used for the diagnosis and management of patients with RD (67–69). Whilst this observation suggests that alterations in tryptophan and kynurenine are not necessarily correlated with CRP elevations, further research is required to determine whether the measurement of these metabolites significantly enhances diagnostic capacity, particularly in early disease, over and above available criteria for RD (70–74). Another interesting observation, in subgroup analysis, was the different association between the reported alterations in tryptophan and kynurenine concentrations in specific types of RD. For example, the SMD of the kynurenine to tryptophan ratio was statistically significant in SLE, pSS, and autoimmune RD type, but not RA, AS, or mixed autoimmune-autoinflammatory RD type. Once again, future studies should investigate whether kynurenine metabolites are useful for the diagnosis and management of specific types of RD. This proposition is supported by the results of an elegant study reporting the utility of kynurenine pathway metabolomics in discriminating clinical subtypes in patients with multiple sclerosis and identifying those at risk of progression (75). Importantly, the absence of significant differences in the effect size between Asian and European studies downplays the potential role of ethnicity as a factor influencing the link between the kynurenine pathway and RD and confirms the results of previous studies investigating this issue (76).

Other significant RD-associated alterations in the kynurenine pathway observed in our study involved the metabolites 3-hydroxykynurenine and quinolinic acid. 3-hydroxykynurenine, derived from kynurenine by kynurenine hydroxylase, has been shown to exert a bi-modal pro-oxidant and antioxidant effect in the central nervous system (77). Further studies have also highlighted the potential for this molecule to bind to α-synuclein and amyloid-beta peptides, with the potential of triggering neuro-inflammatory and neurotoxic processes (78). Quinolinic acid has been extensively investigated as an N-methyl-D-aspartate receptor agonist with pro-oxidant and neurotoxic effects and as a biomarker of neurodegenerative and depressive disorders and inflammatory states (79–83). The results of our systematic review and meta-analysis extend the potential role of 3-hydroxykynurenine and quinolinic acid to the pathophysiology of RD and warrant further research to investigate their local and systemic effects in autoimmune and autoinflammatory conditions, and their role in the reported associations between RD and neuropsychiatric disorders (84–86).

An important additional issue, not investigated in our study, is related to the potential role of dietary factors as well as physical activity in modulating the complex interplay between the kynurenine pathway of tryptophan metabolism and RD. Emerging evidence underscores the role of diet in shaping the kynurenine pathway, thereby affecting the metabolism of tryptophan (87–89). A high-fat diet, for instance, has been shown to modify the flux of metabolites along the kynurenine pathway, suggesting that diet could potentially introduce a confounding element in studies comparing metabolite profiles between RD patients and healthy individuals. Additionally, recent studies have reported that downstream metabolites of kynurenine, particularly kynurenic acid, are increased in muscle biopsies of physically active adults, including after an acute endurance exercise, and associated with cardiorespiratory fitness (90–92). Therefore, future studies should also consider dietary habits and patterns of physical activity as potential factors contributing to the observed variations in the concentrations of tryptophan metabolites. Furthermore, the concept of a comorbidome, referring to the collective presence of multiple comorbid conditions in a patient, introduces another layer of complexity. For example, recent studies have reported that comorbid conditions can interact with the kynurenine pathway and potentially alter the concentrations of tryptophan metabolites (93). Considering the intricate interplay between various medical conditions and metabolic pathways, it is plausible that the presence of a distinct comorbidome might confound the association between RD and tryptophan metabolites in individual patients.

Our systematic review and meta-analysis have several strengths, including the comprehensive assessment of the kynurenine pathway in a wide range of RD types, the study of possible associations between the effect size of between-group differences and clinical and demographic characteristics and analytical methods used, and a rigorous assessment of the risk of bias and the certainty of evidence. A significant limitation is represented by the high between-study heterogeneity observed. However, we were able to identify specific sources of heterogeneity in subgroup analyses for tryptophan (analytical method and matrix used), kynurenine (type of RD and matrix used), kynurenine to tryptophan ratio (analytical method and matrix used). Furthermore, sensitivity analysis ruled out the effect of individual studies on the overall effect size.

In conclusion, this systematic review and meta-analysis has demonstrated the presence of significant alterations in metabolites within the kynurenine pathway of tryptophan metabolism in patients with RD, particularly tryptophan, kynurenine, 3-hydroxykynureine, and quinolinic acid. Further studies, particularly in view of the relatively low certainty of evidence in our analysis, are required to determine the capacity of kynurenine metabolites to identify early and overt types of RD over and above existing clinical criteria and biomarkers with a view to improve the management and quality of life in this complex patient population.

## Data availability statement

The original contributions presented in the study are included in the article/**Supplementary Material**. Further inquiries can be directed to the corresponding author.

## Author contributions

AM: Conceptualization, Methodology, Validation, Visualization, Writing – original draft, Writing – review & editing. AZ: Conceptualization, Data curation, Investigation, Methodology, Validation, Writing – review & editing.

## Supplementary material

Supplementary Figure 1 Sensitivity analysis of the association between tryptophan concentrations and rheumatic disease.

Supplementary Figure 2 Funnel plot of studies investigating associations between tryptophan and rheumatic disease after “trimming-and-filling”.

Supplementary Figure 3 Forest plot of studies investigating tryptophan concentrations in patients and controls according to the presence of autoimmune, mixed autoimmune-autoinflammatory, or autoinflammatory disease.

Supplementary Figure 4 Sensitivity analysis of the association between kynurenine concentrations and rheumatic disease.

Supplementary Figure 5 Funnel plot of studies investigating the association between kynurenine and rheumatic disease after “trimming-and-filling”.

Supplementary Figure 6 Forest plot of studies investigating kynurenine concentrations in patients and controls according to the presence of autoimmune, mixed autoimmune-autoinflammatory, or autoinflammatory disease.

Supplementary Figure 7 Sensitivity analysis of the association between the kynurenine/tryptophan ratio and rheumatic disease.

Supplementary Figure 8 Funnel plot of studies investigating the association between the kynurenine/tryptophan ratio and rheumatic disease after “trimming-and-filling”.

Supplementary Figure 9 Forest plot of studies reporting the kynurenine/tryptophan ratio in patients and controls according to the presence of autoimmune, mixed autoimmune-autoinflammatory, or autoinflammatory diseases.

Supplementary Figure 10 Sensitivity analysis of the association between kynurenic acid concentrations and rheumatic disease.

Supplementary Figure 11 Sensitivity analysis of the association between 3-hydroxyanthranilic acid concentrations and rheumatic disease.

Supplementary Figure 12 Sensitivity analysis of the association between 3-hydroxykynurenine concentrations and rheumatic disease.

Supplementary Figure 13 Sensitivity analysis of the association between quinolinic acid concentrations and rheumatic disease.

## References

[1] Bender DA . Biochemistry of tryptophan in health and disease. Mol Aspects Med (1983) 6(2):101–97. doi: 10.1016/0098-2997(83)90005-5 6371429

[2] Badawy AA . Tryptophan metabolism, disposition and utilization in pregnancy. Biosci Rep (2015) 35(5):e00261. doi: 10.1042/BSR20150197 26381576 PMC4626867

[3] Badawy AA . Kynurenine pathway of tryptophan metabolism: regulatory and functional aspects. Int J Tryptophan Res (2017) 10:1178646917691938. doi: 10.1177/1178646917691938 28469468 PMC5398323

[4] Athnaiel O Ong C Knezevic NN . The role of kynurenine and its metabolites in comorbid chronic pain and depression. Metabolites (2022) 12(10):950. doi: 10.3390/metabo12100950 36295852 PMC9611722

[5] Huang YS Ogbechi J Clanchy FI Williams RO Stone TW . IDO and kynurenine metabolites in peripheral and CNS disorders. Front Immunol (2020) 11:388. doi: 10.3389/fimmu.2020.00388 32194572 PMC7066259

[6] Chen LM Bao CH Wu Y Liang SH Wang D Wu LY . Tryptophan-kynurenine metabolism: a link between the gut and brain for depression in inflammatory bowel disease. J Neuroinflamm (2021) 18(1):135. doi: 10.1186/s12974-021-02175-2 PMC8204445 34127024

[7] Comai S Bertazzo A Brughera M Crotti S . Tryptophan in health and disease. Adv Clin Chem (2020) 95:165–218. doi: 10.1016/bs.acc.2019.08.005 32122523

[8] Wang Q Liu D Song P Zou MH . Tryptophan-kynurenine pathway is dysregulated in inflammation, and immune activation. Front Biosci (Landmark Ed) (2015) 20(7):1116–43. doi: 10.2741/4363 PMC4911177 25961549

[9] Calle E Gómez-Puerta JA . The spectrum of rheumatic diseases. Surgery in rheumatic and musculoskeletal disease. Handb Syst Autoimmune Dis (2018) 15:1–13. doi: 10.1016/B978-0-444-63887-8.00001-3

[10] McGonagle D McDermott MF . A proposed classification of the immunological diseases. PloS Med (2006) 3(8):e297. doi: 10.1371/journal.pmed.0030297 16942393 PMC1564298

[11] Moutsopoulos HM . Autoimmune rheumatic diseases: One or many diseases? J Transl Autoimmun (2021) 4:100129. doi: 10.1016/j.jtauto.2021.100129 35005593 PMC8716565

[12] Mallen CD Helliwell T Scott IC . How can primary care physicians enhance the early diagnosis of rheumatic diseases? Expert Rev Clin Immunol (2018) 14(3):171–3. doi: 10.1080/1744666X.2018.1429919 29338450

[13] Mohan C Assassi S . Biomarkers in rheumatic diseases: how can they facilitate diagnosis and assessment of disease activity? BMJ (2015) 351:h5079. doi: 10.1136/bmj.h5079 26612523 PMC6882504

[14] Guma M Tiziani S Firestein GS . Metabolomics in rheumatic diseases: desperately seeking biomarkers. Nat Rev Rheumatol (2016) 12(5):269–81. doi: 10.1038/nrrheum.2016.1 PMC4963238 26935283

[15] Moola S Munn Z Tufanaru C Aromataris E Sears K Sfetcu R . Systematic reviews of etiology and risk. In: Aromataris E Munn Z , editors. Joanna Briggs Institute Reviewer’s Manual. Adelaide, Australia: Johanna Briggs Institute (2017).

[16] Balshem H Helfand M Schunemann HJ Oxman AD Kunz R Brozek J . GRADE guidelines: 3. Rating the quality of evidence. J Clin Epidemiol (2011) 64(4):401–6. doi: 10.1016/j.jclinepi.2010.07.015 21208779

[17] Page MJ McKenzie JE Bossuyt PM Boutron I Hoffmann TC Mulrow CD . The PRISMA 2020 statement: an updated guideline for reporting systematic reviews. BMJ (2021) 372:n71. doi: 10.1136/bmj.n71 33782057 PMC8005924

[18] Wan X Wang W Liu J Tong T . Estimating the sample mean and standard deviation from the sample size, median, range and/or interquartile range. BMC Med Res Methodol (2014) 14:135. doi: 10.1186/1471-2288-14-135 25524443 PMC4383202

[19] Hozo SP Djulbegovic B Hozo I . Estimating the mean and variance from the median, range, and the size of a sample. BMC Med Res Methodol (2005) 5:13. doi: 10.1186/1471-2288-5-13 15840177 PMC1097734

[20] Higgins JP Thompson SG . Quantifying heterogeneity in a meta-analysis. Stat Med (2002) 21(11):1539–58. doi: 10.1002/sim.1186 12111919

[21] Higgins JP Thompson SG Deeks JJ Altman DG . Measuring inconsistency in meta-analyses. BMJ (2003) 327(7414):557–60. doi: 10.1136/bmj.327.7414.557 PMC192859 12958120

[22] Tobias A . Assessing the influence of a single study in the meta-analysis estimate. Stata Tech Bull (1999) 47:15–7.

[23] Begg CB Mazumdar M . Operating characteristics of a rank correlation test for publication bias. Biometrics (1994) 50(4):1088–101. doi: 10.2307/2533446 7786990

[24] Sterne JA Egger M . Funnel plots for detecting bias in meta-analysis: guidelines on choice of axis. J Clin Epidemiol (2001) 54(10):1046–55. doi: 10.1016/s0895-4356(01)00377-8 11576817

[25] Duval S Tweedie R . Trim and fill: A simple funnel-plot-based method of testing and adjusting for publication bias in meta-analysis. Biometrics (2000) 56(2):455–63. doi: 10.1111/j.0006-341x.2000.00455.x 10877304

[26] Csipo I Czirjak L Szanto S Szerafin L Sipka S Szegedi G . Decreased serum tryptophan and elevated neopterin levels in systemic sclerosis. Clin Exp Rheumatol (1995) 13(2):269–70. 7656478

[27] Widner B Sepp N Kowald E Ortner U Wirleitner B Fritsch P . Enhanced tryptophan degradation in systemic lupus erythematosus. Immunobiology (2000) 201(5):621–30. doi: 10.1016/S0171-2985(00)80079-0 10834318

[28] Schroecksnadel K Kaser S Ledochowski M Neurauter G Mur E Herold M . Increased degradation of tryptophan in blood of patients with rheumatoid arthritis. J Rheumatol (2003) 30(9):1935–9. 12966593

[29] Pertovaara M Raitala A Uusitalo H Pukander J Helin H Oja SS . Mechanisms dependent on tryptophan catabolism regulate immune responses in primary Sjogren's syndrome. Clin Exp Immunol (2005) 142(1):155–61. doi: 10.1111/j.1365-2249.2005.02889.x PMC1809473 16178870

[30] Xiang ZY Tang AG Ren YP Zhou QX Luo XB . Simultaneous determination of serum tryptophan metabolites in patients with systemic lupus erythematosus by high performance liquid chromatography with fluorescence detection. Clin Chem Lab Med (2010) 48(4):513–7. doi: 10.1515/CCLM.2010.105 20187853

[31] Ozkan Y Mete G Sepici-Dincel A Sepici V Simsek B . Tryptophan degradation and neopterin levels in treated rheumatoid arthritis patients. Clin Rheumatol (2012) 31(1):29–34. doi: 10.1007/s10067-011-1767-5 21556779

[32] Lood C Tyden H Gullstrand B Klint C Wenglen C Nielsen CT . Type I interferon-mediated skewing of the serotonin synthesis is associated with severe disease in systemic lupus erythematosus. PloS One (2015) 10(4):e0125109. doi: 10.1371/journal.pone.0125109 25897671 PMC4405357

[33] Maria NI van Helden-Meeuwsen CG Brkic Z Paulissen SM Steenwijk EC Dalm VA . Association of increased treg cell levels with elevated indoleamine 2,3-dioxygenase activity and an imbalanced kynurenine pathway in interferon-positive primary Sjogren's syndrome. Arthritis Rheumatol (2016) 68(7):1688–99. doi: 10.1002/art.39629 26866723

[34] Smolenska Z Smolenski RT Zdrojewski Z . Plasma concentrations of amino acid and nicotinamide metabolites in rheumatoid arthritis–potential biomarkers of disease activity and drug treatment. Biomarkers (2016) 21(3):218–24. doi: 10.3109/1354750X.2015.1130746 26811910

[35] Akesson K Pettersson S Stahl S Surowiec I Hedenstrom M Eketjall S . Kynurenine pathway is altered in patients with SLE and associated with severe fatigue. Lupus Sci Med (2018) 5(1):e000254. doi: 10.1136/lupus-2017-000254 29868176 PMC5976103

[36] Urbaniak B Plewa S Klupczynska A Sikorska D Samborski W Kokot ZJ . Serum free amino acid levels in rheumatoid arthritis according to therapy and physical disability. Cytokine (2019) 113:332–9. doi: 10.1016/j.cyto.2018.10.002 30337216

[37] Smolenska Z Zabielska-Kaczorowska M Wojteczek A Kutryb-Zajac B Zdrojewski Z . Metabolic pattern of systemic sclerosis: association of changes in plasma concentrations of amino acid-related compounds with disease presentation. Front Mol Biosci (2020) 7:585161. doi: 10.3389/fmolb.2020.585161 33195431 PMC7593705

[38] Zhou Y Zhang X Chen R Han S Liu Y Liu X . Serum amino acid metabolic profiles of ankylosing spondylitis by targeted metabolomics analysis. Clin Rheumatol (2020) 39(8):2325–36. doi: 10.1007/s10067-020-04974-z 32130577

[39] Anderson EW Fishbein J Hong J Roeser J Furie RA Aranow C . Quinolinic acid, a kynurenine/tryptophan pathway metabolite, associates with impaired cognitive test performance in systemic lupus erythematosus. Lupus Sci Med (2021) 8(1):e000559. doi: 10.1136/lupus-2021-000559 34686589 PMC8543639

[40] Eryavuz Onmaz D Sivrikaya A Isik K Abusoglu S Albayrak Gezer I Humeyra Yerlikaya F . Altered kynurenine pathway metabolism in patients with ankylosing spondylitis. Int Immunopharmacol (2021) 99:108018. doi: 10.1016/j.intimp.2021.108018 34358860

[41] Kor A Erten Ş Yurt EF Dogan İ Apaydin H Aserdar M . Clinical significance of plasma tryptophan, kynurenine, and kynurenine/tryptophan ratio in rheumatoid arthritis patients. Egypt Rheumatol (2022) 44(4):367–71. doi: 10.1016/j.ejr.2022.07.005

[42] Eryavuz Onmaz D Tezcan D Abusoglu S Sivrikaya A Kuzu M Yerlikaya FH . Elevated serum levels of kynurenine pathway metabolites in patients with Behcet disease. Amino Acids (2022) 54(6):877–87. doi: 10.1007/s00726-022-03170-4 35604497

[43] Pellicano C Vaiarello V Colalillo A Gigante A Iannazzo F Rosato E . Role of kinurenic acid in the systemic sclerosis renal involvement. Clin Exp Med (2022) (5):1713–9. doi: 10.1007/s10238-022-00962-6 36436115

[44] Apaydin H Koca Bicer C Feyza Yurt E Abdulkadir Serdar M Dogan I Erten S . Elevated kynurenine levels in patients with primary Sjogren's syndrome. Lab Med (2023) 54(2):166–72. doi: 10.1093/labmed/lmac084 36053233

[45] Jeon C Jang Y Lee SH Weon S Park H Lee S . Abnormal kynurenine level contributes to the pathological bone features of ankylosing spondylitis. Int Immunopharmacol (2023) 118:110132. doi: 10.1016/j.intimp.2023.110132 37023698

[46] Eryavuz Onmaz D Tezcan D Abusoglu S Sak F Humeyra Yerlikaya F Yilmaz S . Impaired kynurenine metabolism in patients with primary Sjogren's syndrome. Clin Biochem (2023) 114:1–10. doi: 10.1016/j.clinbiochem.2023.01.007 36681140

[47] Park Y Lee JJ Koh JH Kim MJ Park SH Kwok SK . Kynurenine pathway can be a potential biomarker of fatigue in primary Sjogren's syndrome. Clin Exp Rheumatol (2023). doi: 10.55563/clinexprheumatol/cp4st9 (In press). 36826785

[48] Tezcan D Onmaz DE Sivrikaya A Korez MK Hakbilen S Gulcemal S . Kynurenine pathway of tryptophan metabolism in patients with familial Mediterranean fever. Mod Rheumatol (2023) 33(2):398–407. doi: 10.1093/mr/roac016 35139221

[49] Yurt EF Bicer C Serdar MA Akan S Erten S . Accelerated kynurenine pathway downregulates immune activation in patients with axial spondyloarthritis. Cytokine (2023) 169:156247. doi: 10.1016/j.cyto.2023.156247 37295242

[50] Cohen J . Statistical power analysis. Curr Dir Psychol Sci (1992) 1(3):98–101. doi: 10.1111/1467-8721.ep10768783

[51] Newton A McCann L Huo L Liu A . Kynurenine pathway regulation at its critical junctions with fluctuation of tryptophan. Metabolites (2023) 13(4):500. doi: 10.3390/metabo13040500 37110158 PMC10143591

[52] Pfefferkorn ER Rebhun S Eckel M . Characterization of an indoleamine 2,3-dioxygenase induced by gamma-interferon in cultured human fibroblasts. J Interferon Res (1986) 6(3):267–79. doi: 10.1089/jir.1986.6.267 2427623

[53] Ozaki Y Edelstein MP Duch DS . The actions of interferon and antiinflammatory agents of induction of indoleamine 2,3-dioxygenase in human peripheral blood monocytes. Biochem Biophys Res Commun (1987) 144(3):1147–53. doi: 10.1016/0006-291x(87)91431-8 3107562

[54] Chizzolini C Dayer JM Miossec P . Cytokines in chronic rheumatic diseases: is everything lack of homeostatic balance? Arthritis Res Ther (2009) 11(5):246. doi: 10.1186/ar2767 19849823 PMC2787274

[55] Isomaki P Punnonen J . Pro- and anti-inflammatory cytokines in rheumatoid arthritis. Ann Med (1997) 29(6):499–507. doi: 10.3109/07853899709007474 9562516

[56] Markovics A Rosenthal KS Mikecz K Carambula RE Ciemielewski JC Zimmerman DH . Restoring the balance between pro-inflammatory and anti-inflammatory cytokines in the treatment of rheumatoid arthritis: new insights from animal models. Biomedicines (2021) 10(1):44. doi: 10.3390/biomedicines10010044 35052724 PMC8772713

[57] Forstermann U Sessa WC . Nitric oxide synthases: regulation and function. Eur Heart J (2012) 33(7):829–37. doi: 10.1093/eurheartj/ehr304 PMC3345541 21890489

[58] Bian K Murad F . Nitric oxide (NO)–biogeneration, regulation, and relevance to human diseases. Front Biosci (2003) 8:d264–78. doi: 10.2741/997 12456375

[59] Mangoni AA Tommasi S Sotgia S Zinellu A Paliogiannis P Piga M . Asymmetric dimethylarginine: a key player in the pathophysiology of endothelial dysfunction, vascular inflammation and atherosclerosis in rheumatoid arthritis? Curr Pharm Des (2021) 27(18):2131–40. doi: 10.2174/1381612827666210106144247 33413061

[60] Erre GL Buscetta G Paliogiannis P Mangoni AA Carru C Passiu G . Coronary flow reserve in systemic rheumatic diseases: a systematic review and meta-analysis. Rheumatol Int (2018) 38(7):1179–90. doi: 10.1007/s00296-018-4039-8 29732488

[61] Erre GL Piga M Fedele AL Mura S Piras A Cadoni ML . Prevalence and determinants of peripheral microvascular endothelial dysfunction in rheumatoid arthritis patients: A multicenter cross-sectional study. Mediators Inflamm (2018) 2018:6548715. doi: 10.1155/2018/6548715 29483841 PMC5816852

[62] Erre GL Mangoni AA Castagna F Paliogiannis P Carru C Passiu G . Meta-analysis of asymmetric dimethylarginine concentrations in rheumatic diseases. Sci Rep (2019) 9(1):5426. doi: 10.1038/s41598-019-41994-5 30932011 PMC6443686

[63] Luczak A Madej M Kasprzyk A Doroszko A . Role of the eNOS uncoupling and the nitric oxide metabolic pathway in the pathogenesis of autoimmune rheumatic diseases. Oxid Med Cell Longev (2020) 2020:1417981. doi: 10.1155/2020/1417981 32351667 PMC7174952

[64] Hedar AM Stradner MH Roessler A Goswami N . Autoimmune rheumatic diseases and vascular function: the concept of autoimmune atherosclerosis. J Clin Med (2021) 10(19):4427. doi: 10.3390/jcm10194427 34640445 PMC8509415

[65] Drosos GC Vedder D Houben E Boekel L Atzeni F Badreh S . EULAR recommendations for cardiovascular risk management in rheumatic and musculoskeletal diseases, including systemic lupus erythematosus and antiphospholipid syndrome. Ann Rheum Dis (2022) 81(6):768–79. doi: 10.1136/annrheumdis-2021-221733 35110331

[66] Mackey RH Kuller LH Moreland LW . Update on cardiovascular disease risk in patients with rheumatic diseases. Rheum Dis Clin North Am (2018) 44(3):475–87. doi: 10.1016/j.rdc.2018.03.006 30001787

[67] Szalai AJ . C-reactive protein (CRP) and autoimmune disease: facts and conjectures. Clin Dev Immunol (2004) 11(3-4):221–6. doi: 10.1080/17402520400001751 PMC2486333 15559367

[68] Pope JE Choy EH . C-reactive protein and implications in rheumatoid arthritis and associated comorbidities. Semin Arthritis Rheumatol (2021) 51(1):219–29. doi: 10.1016/j.semarthrit.2020.11.005 33385862

[69] Hilliquin P . Biological markers in inflammatory rheumatic diseases. Cell Mol Biol (Noisy-le-grand) (1995) 41(8):993–1006. 8747080

[70] Shmerling RH . Diagnostic tests for rheumatic disease: clinical utility revisited. South Med J (2005) 98(7):704–11; quiz 12-3, 28. doi: 10.1097/01.smj.0000171073.07875.c5 16108239

[71] Allen A Carville S McKenna F Guideline Development G . Diagnosis and management of rheumatoid arthritis in adults: summary of updated NICE guidance. BMJ (2018) 362:k3015. doi: 10.1136/bmj.k3015 30076129

[72] Aggarwal R Ringold S Khanna D Neogi T Johnson SR Miller A . Distinctions between diagnostic and classification criteria? Arthritis Care Res (Hoboken) (2015) 67(7):891–7. doi: 10.1002/acr.22583 PMC4482786 25776731

[73] Kwiatkowska B Raciborski F Klak A Maslinska M Gryglewicz J . Early diagnosis of rheumatic diseases: an evaluation of the present situation and proposed changes. Reumatologia (2015) 53(1):3–8. doi: 10.5114/reum.2015.50550 27407218 PMC4847309

[74] Aringer M Johnson SR . Classifying and diagnosing systemic lupus erythematosus in the 21st century. Rheumatol (Oxford) (2020) 59(Suppl5):v4–v11. doi: 10.1093/rheumatology/keaa379 PMC7719035 33280013

[75] Lim CK Bilgin A Lovejoy DB Tan V Bustamante S Taylor BV . Kynurenine pathway metabolomics predicts and provides mechanistic insight into multiple sclerosis progression. Sci Rep (2017) 7:41473. doi: 10.1038/srep41473 28155867 PMC5290739

[76] Badawy AA Dougherty DM . Assessment of the human kynurenine pathway: comparisons and clinical implications of ethnic and gender differences in plasma tryptophan, kynurenine metabolites, and enzyme expressions at baseline and after acute tryptophan loading and depletion. Int J Tryptophan Res (2016) 9:31–49. doi: 10.4137/IJTR.S38189 27547036 PMC4981220

[77] Colin-Gonzalez AL Maldonado PD Santamaria A . 3-Hydroxykynurenine: an intriguing molecule exerting dual actions in the central nervous system. Neurotoxicology (2013) 34:189–204. doi: 10.1016/j.neuro.2012.11.007 23219925

[78] Capucciati A Galliano M Bubacco L Zecca L Casella L Monzani E . Neuronal proteins as targets of 3-hydroxykynurenine: implications in neurodegenerative diseases. ACS Chem Neurosci (2019) 10(8):3731–9. doi: 10.1021/acschemneuro.9b00265 31298828

[79] Hestad K Alexander J Rootwelt H Aaseth JO . The role of tryptophan dysmetabolism and quinolinic acid in depressive and neurodegenerative diseases. Biomolecules (2022) 12(7):998. doi: 10.3390/biom12070998 35883554 PMC9313172

[80] Parasram K . Phytochemical treatments target kynurenine pathway induced oxidative stress. Redox Rep (2018) 23(1):25–8. doi: 10.1080/13510002.2017.1343223 PMC6748679 28651456

[81] Geronimo-Olvera C Tristan-Lopez L Martinez-Lazcano JC Garcia-Lara L Sanchez-Mendoza A Morales-Martinez A . Striatal protection in nNOS knock-out mice after quinolinic acid-induced oxidative damage. Neurochem Res (2019) 44(2):421–7. doi: 10.1007/s11064-018-2688-3 30523577

[82] Behan WM McDonald M Darlington LG Stone TW . Oxidative stress as a mechanism for quinolinic acid-induced hippocampal damage: protection by melatonin and deprenyl. Br J Pharmacol (1999) 128(8):1754–60. doi: 10.1038/sj.bjp.0702940 PMC1571800 10588931

[83] Saade MC Clark AJ Parikh SM . States of quinolinic acid excess in urine: A systematic review of human studies. Front Nutr (2022) 9:1070435. doi: 10.3389/fnut.2022.1070435 36590198 PMC9800835

[84] Olah C Schwartz N Denton C Kardos Z Putterman C Szekanecz Z . Cognitive dysfunction in autoimmune rheumatic diseases. Arthritis Res Ther (2020) 22(1):78. doi: 10.1186/s13075-020-02180-5 32293528 PMC7158026

[85] Sundquist K Li X Hemminki K Sundquist J . Subsequent risk of hospitalization for neuropsychiatric disorders in patients with rheumatic diseases: a nationwide study from Sweden. Arch Gen Psychiatry (2008) 65(5):501–7. doi: 10.1001/archpsyc.65.5.501 18458201

[86] Marrie RA Hitchon CA Walld R Patten SB Bolton JM Sareen J . Increased burden of psychiatric disorders in rheumatoid arthritis. Arthritis Care Res (Hoboken) (2018) 70(7):970–8. doi: 10.1002/acr.23539 PMC6033023 29438604

[87] Francis HM Stevenson RJ Tan LSY Ehrenfeld L Byeon S Attuquayefio T . Kynurenic acid as a biochemical factor underlying the association between Western-style diet and depression: A cross-sectional study. Front Nutr (2022) 9:945538. doi: 10.3389/fnut.2022.945538 36299996 PMC9589270

[88] Sun P Wang M Liu YX Li L Chai X Zheng W . High-fat diet-disturbed gut microbiota-colonocyte interactions contribute to dysregulating peripheral tryptophan-kynurenine metabolism. Microbiome (2023) 11(1):154. doi: 10.1186/s40168-023-01606-x 37468922 PMC10355067

[89] Damiani G Pacifico A Scoditti E di Gregorio S Del Fabbro M Cozzolino C . Circadian oscillations of minimal erythema dose (MED) are also influenced by diet in patients with psoriasis: A chronomedical study. Dermatol Ther (Heidelb) (2023) 13(10):2229–46. doi: 10.1007/s13555-023-00987-z PMC10539244 37573289

[90] Joisten N Kummerhoff F Koliamitra C Schenk A Walzik D Hardt L . Exercise and the Kynurenine pathway: Current state of knowledge and results from a randomized cross-over study comparing acute effects of endurance and resistance training. Exerc Immunol Rev (2020) 26:24–42. 32139353

[91] Saran T Turska M Kocki T Zawadka M Zielinski G Turski WA . Effect of 4-week physical exercises on tryptophan, kynurenine and kynurenic acid content in human sweat. Sci Rep (2021) 11(1):11092. doi: 10.1038/s41598-021-90616-6 34045580 PMC8160349

[92] Hinkley JM Yu GX Standley RA Distefano G Tolstikov V Narain NR . Exercise and ageing impact the kynurenine/tryptophan pathway and acylcarnitine metabolite pools in skeletal muscle of older adults. J Physiol (2023) 601(11):2165–88. doi: 10.1113/JP284142 PMC10278663 36814134

[93] Buja A Miatton A Cozzolino C Brazzale AR Lo Bue R Mercuri SR . The prevalent comorbidome at the onset of psoriasis diagnosis. Dermatol Ther (Heidelb) (2023) 13(9):2093–105. doi: 10.1007/s13555-023-00986-0 PMC10442308 37542678

