# Supplementary figures and images for: A systematic review and meta-analysis of the kynurenine pathway of tryptophan metabolism in rheumatic diseases
Source: Front Immunol. 2023 Oct 23;14:1257159. doi: 10.3389/fimmu.2023.1257159 (PMC10626995; doi:10.3389/fimmu.2023.1257159)

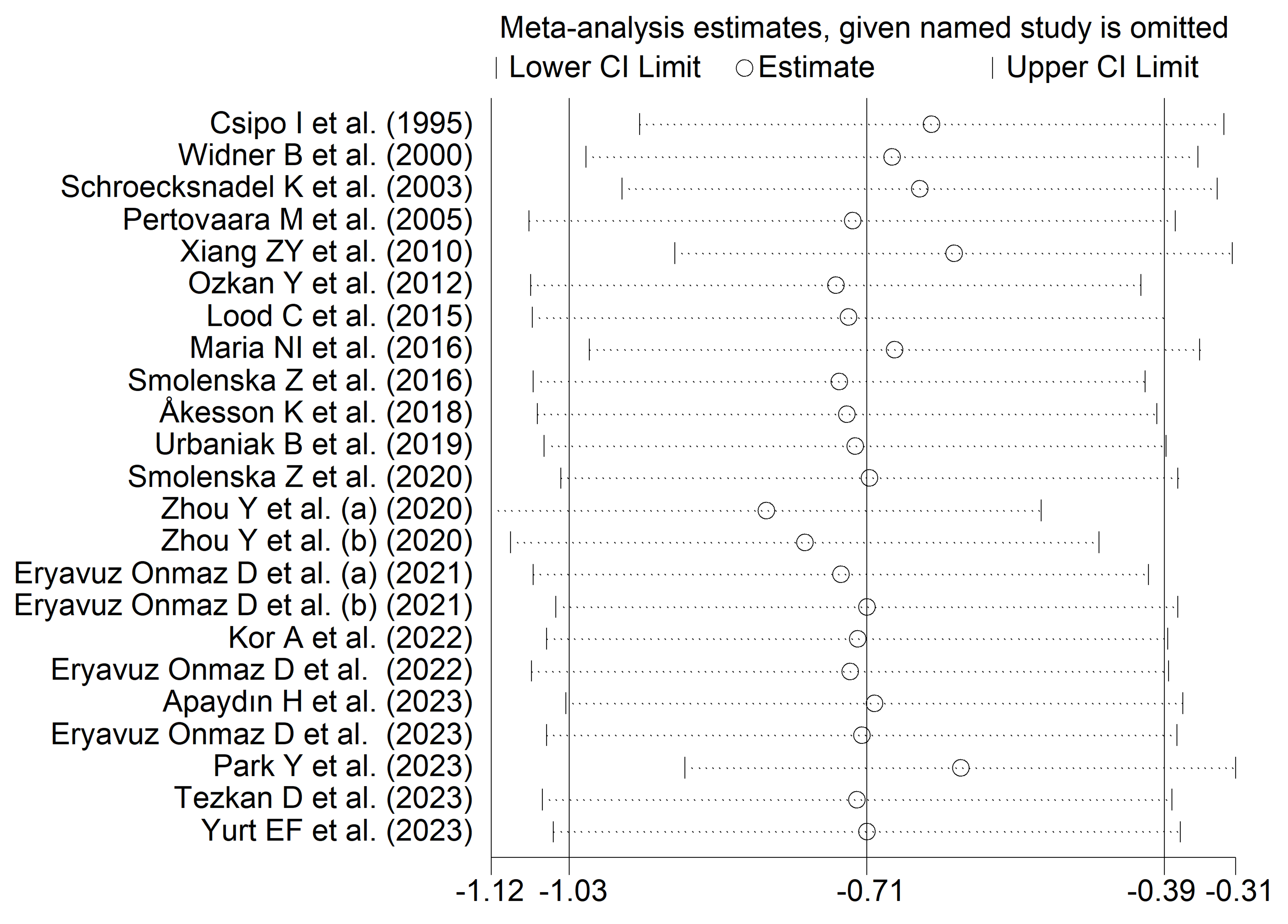

Supplement: Supplementary Figure 1 — Sensitivity analysis of the association between tryptophan concentrations and rheumatic disease. [file Image_1.tif]

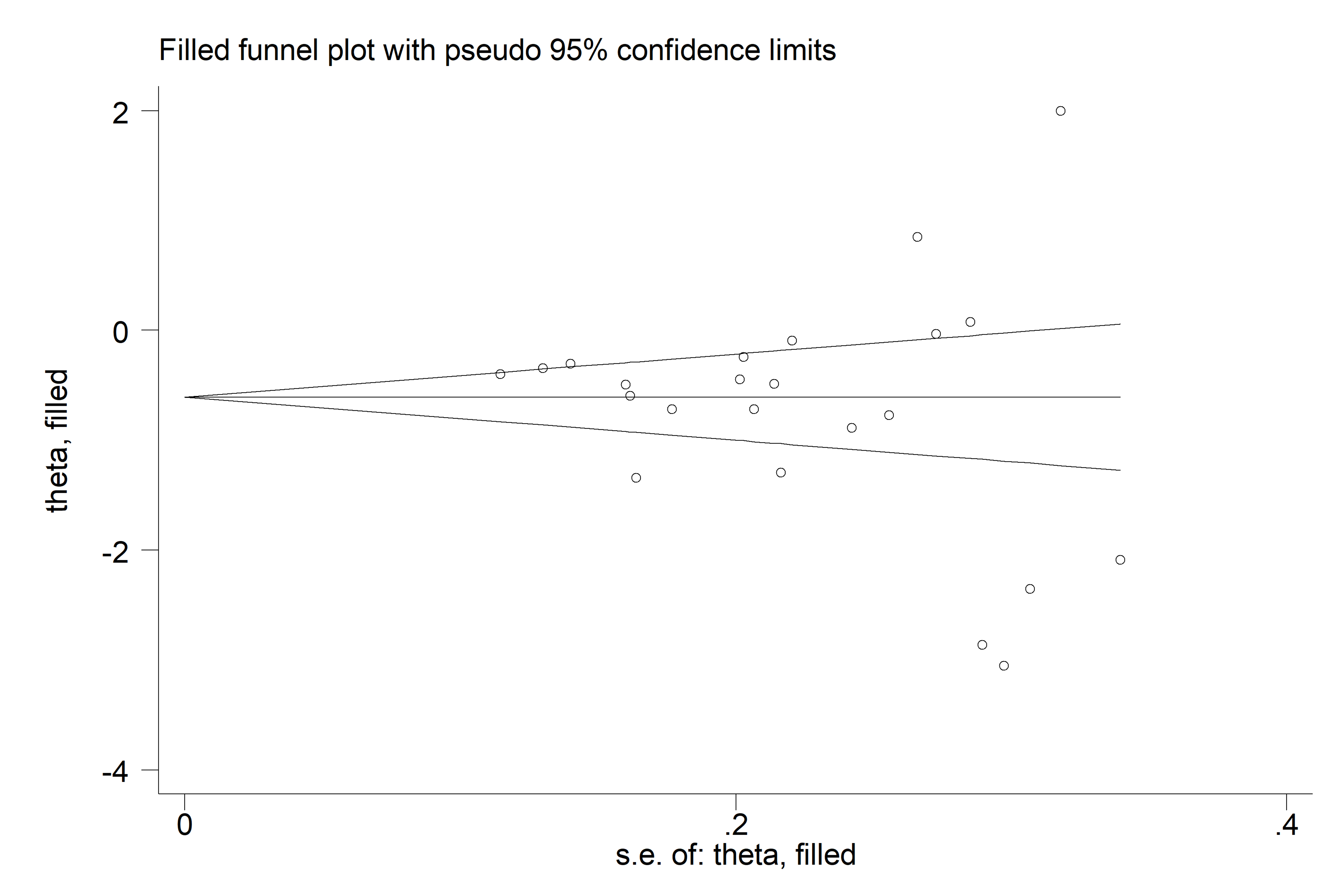

Supplement: Supplementary Figure 2 — Funnel plot of studies investigating associations between tryptophan and rheumatic disease after “trimming-and-filling”. [file Image_2.tif]

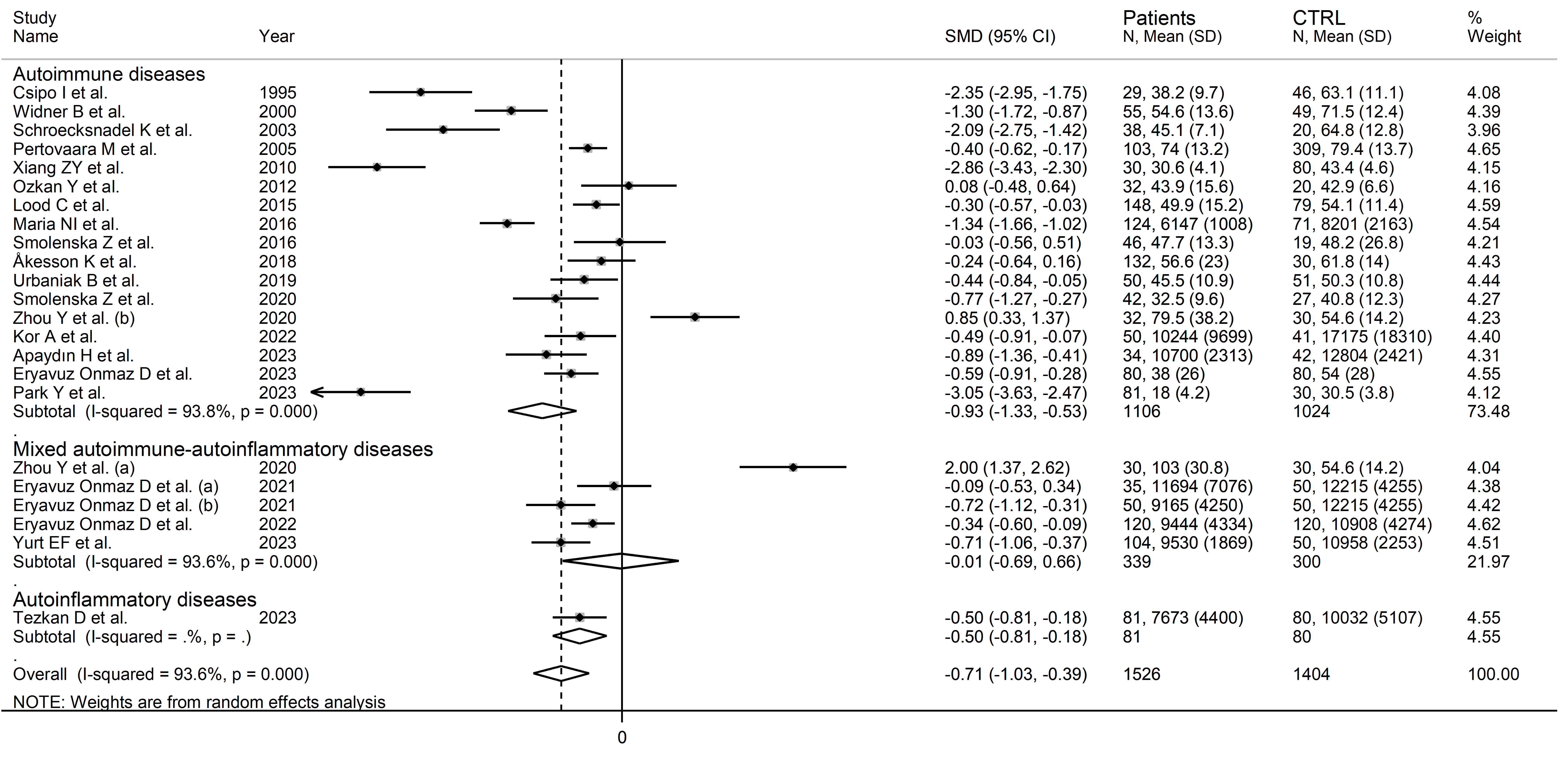

Supplement: Supplementary Figure 3 — Forest plot of studies investigating tryptophan concentrations in patients and controls according to the presence of autoimmune, mixed autoimmune-autoinflammatory, or autoinflammatory disease. [file Image_3.tif]

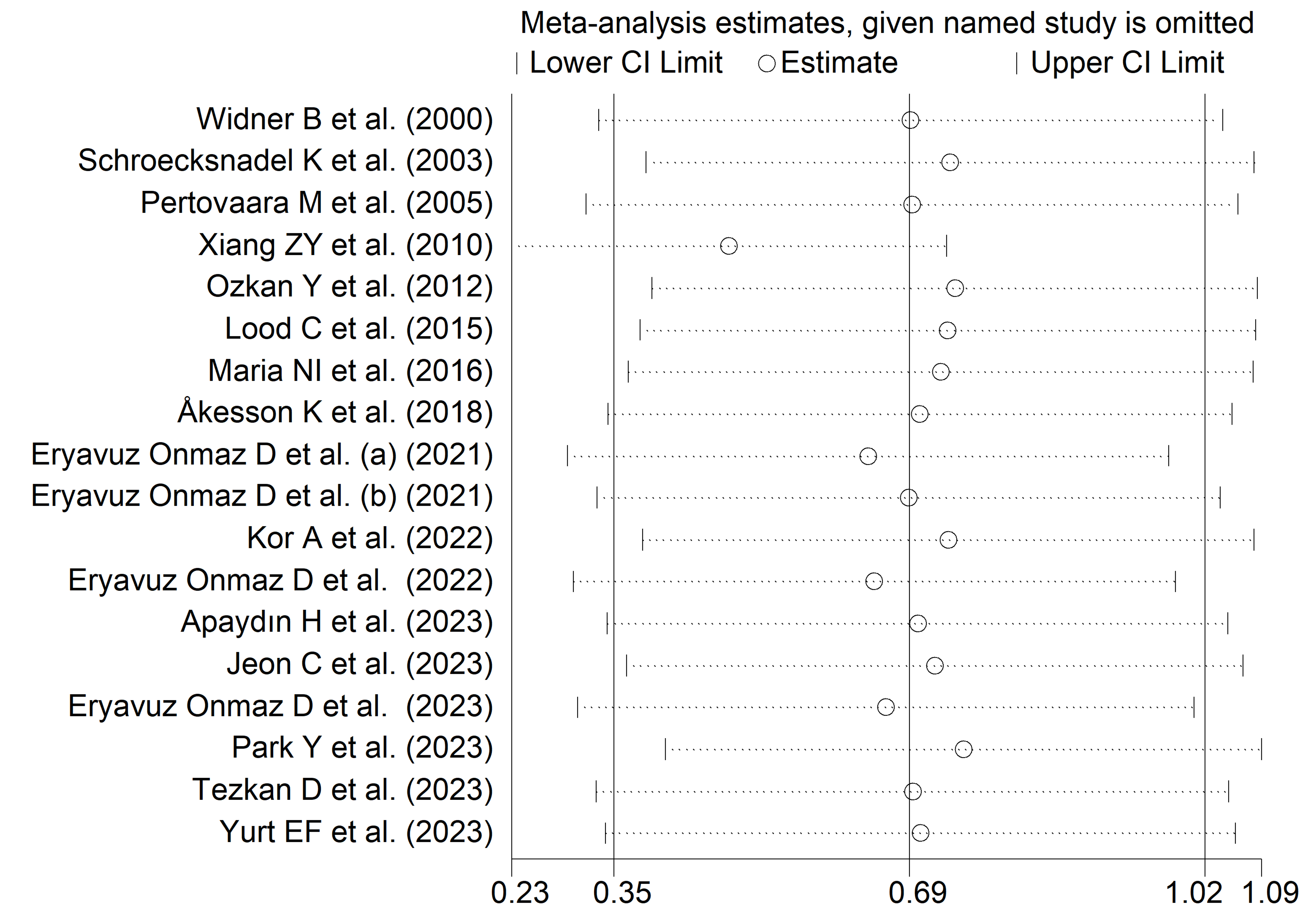

Supplement: Supplementary Figure 4 — Sensitivity analysis of the association between kynurenine concentrations and rheumatic disease. [file Image_4.tif]

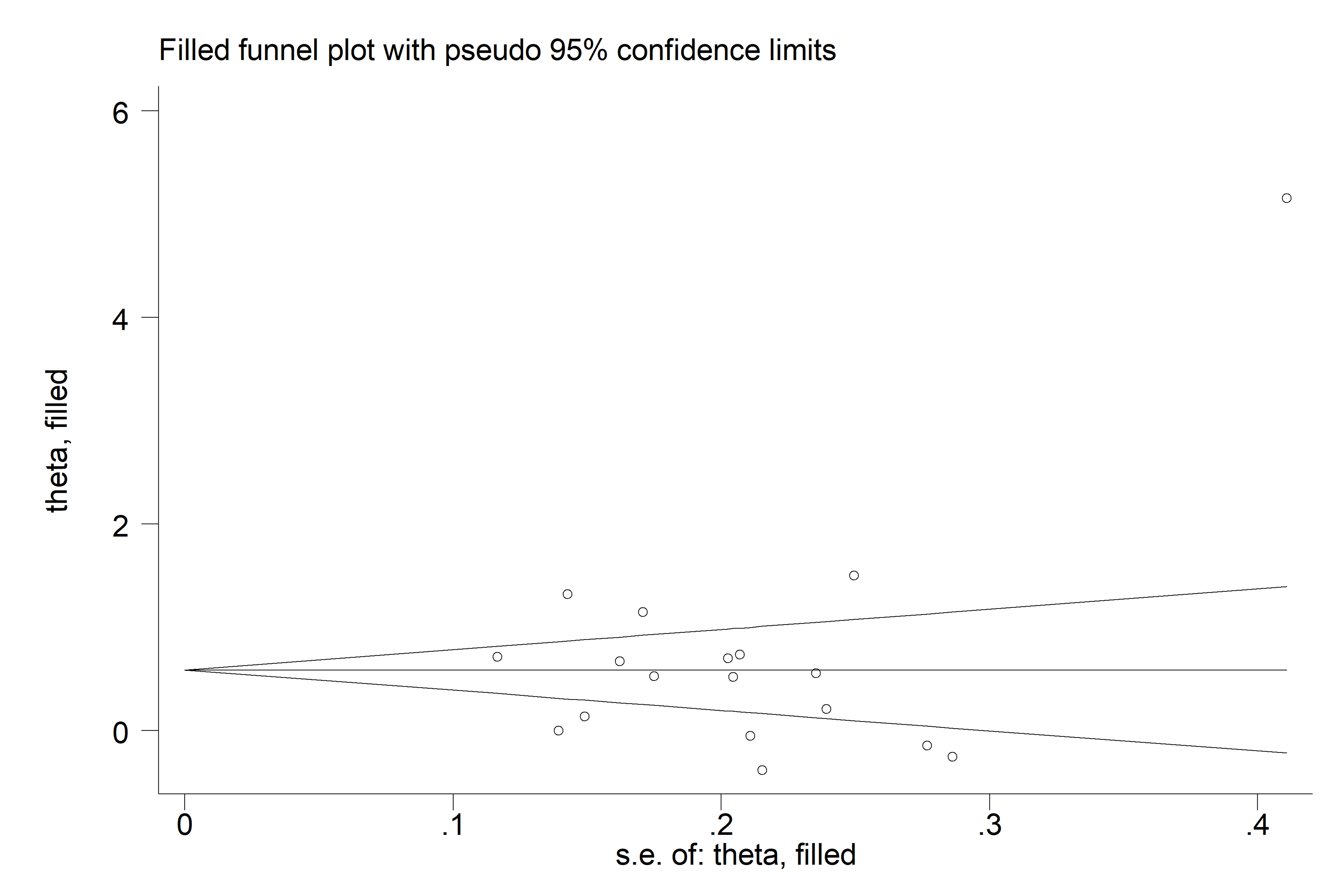

Supplement: Supplementary Figure 5 — Funnel plot of studies investigating the association between kynurenine and rheumatic disease after “trimming-and-filling”. [file Image_5.tif]

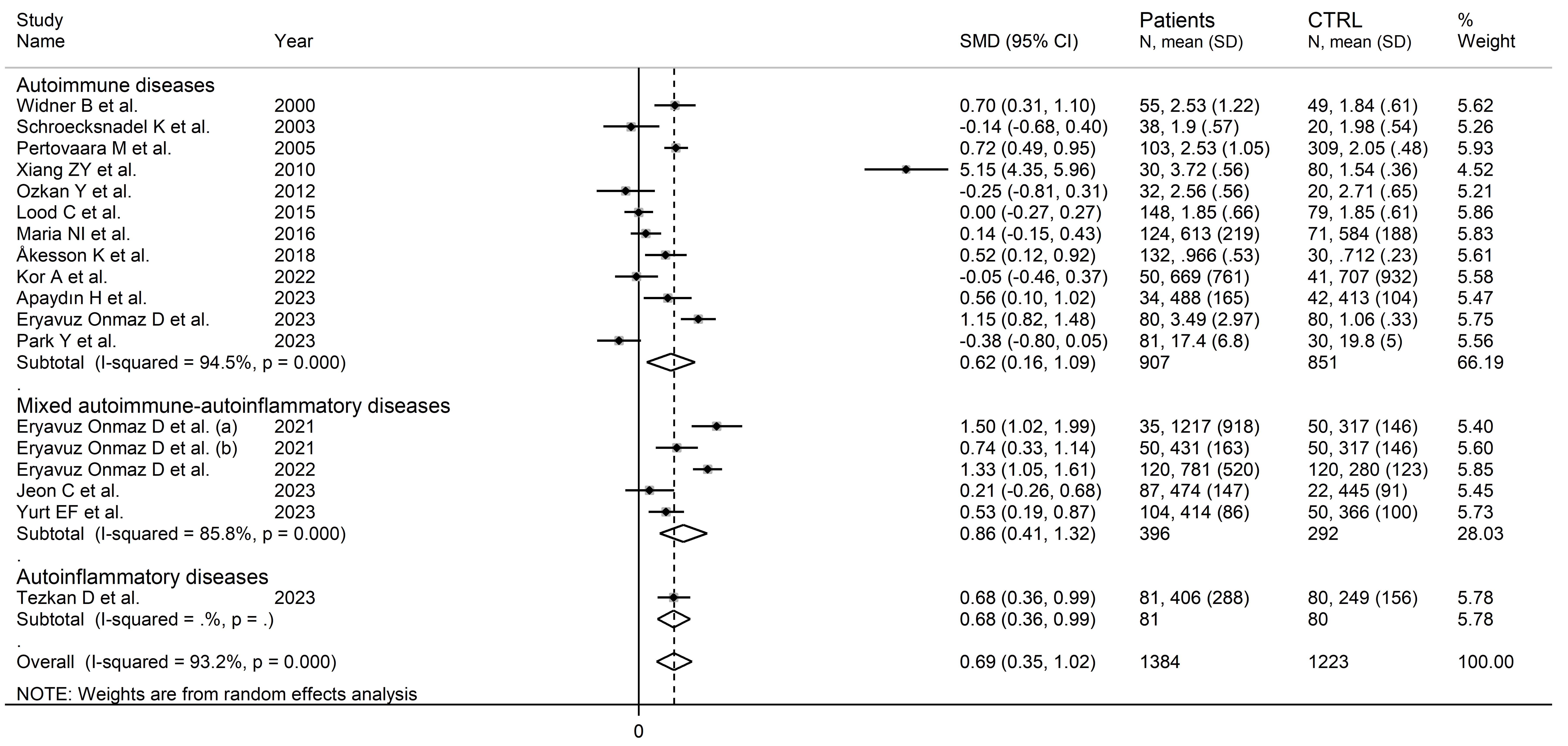

Supplement: Supplementary Figure 6 — Forest plot of studies investigating kynurenine concentrations in patients and controls according to the presence of autoimmune, mixed autoimmune-autoinflammatory, or autoinflammatory disease. [file Image_6.tif]

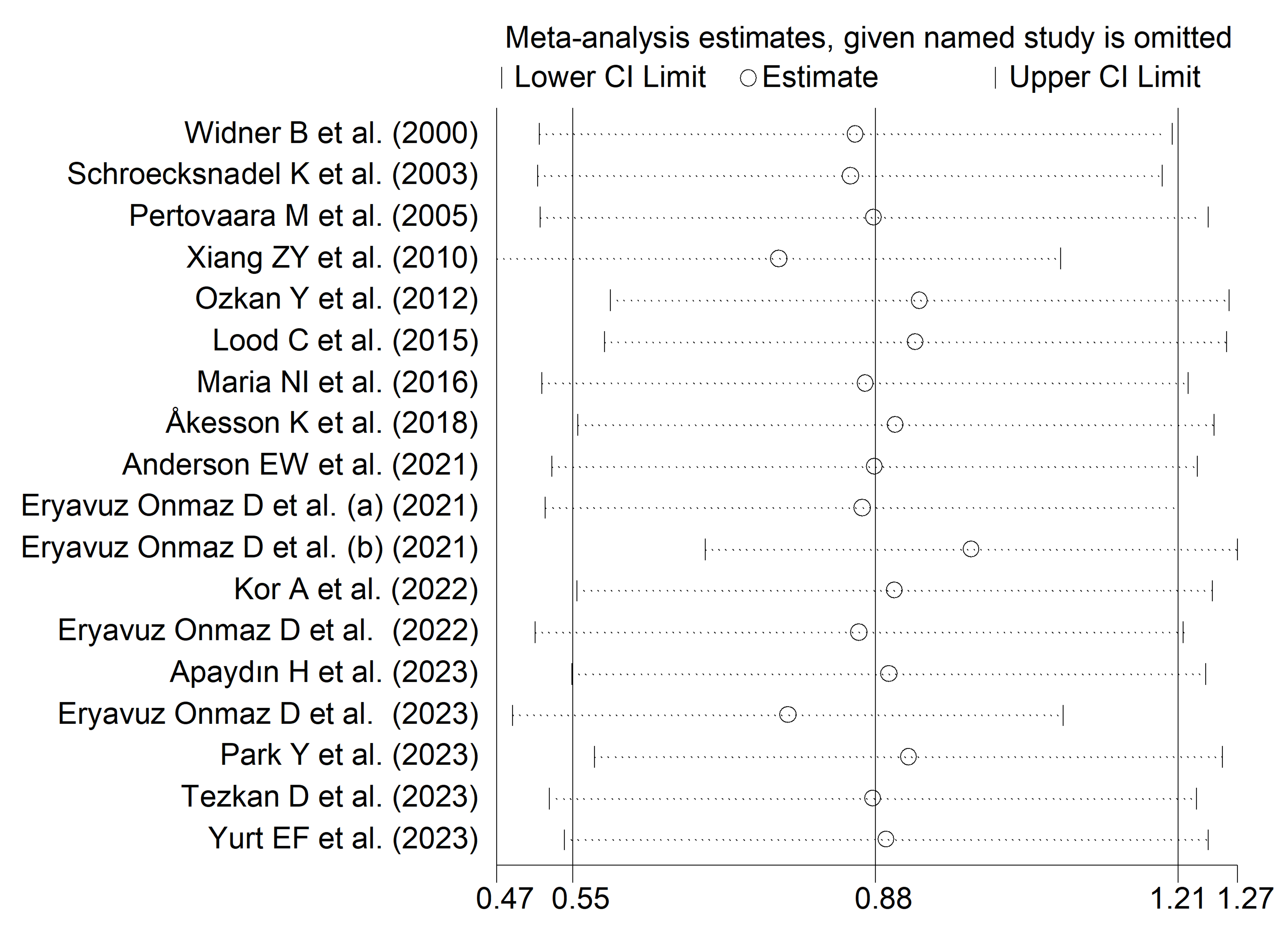

Supplement: Supplementary Figure 7 — Sensitivity analysis of the association between the kynurenine/tryptophan ratio and rheumatic disease. [file Image_7.tif]

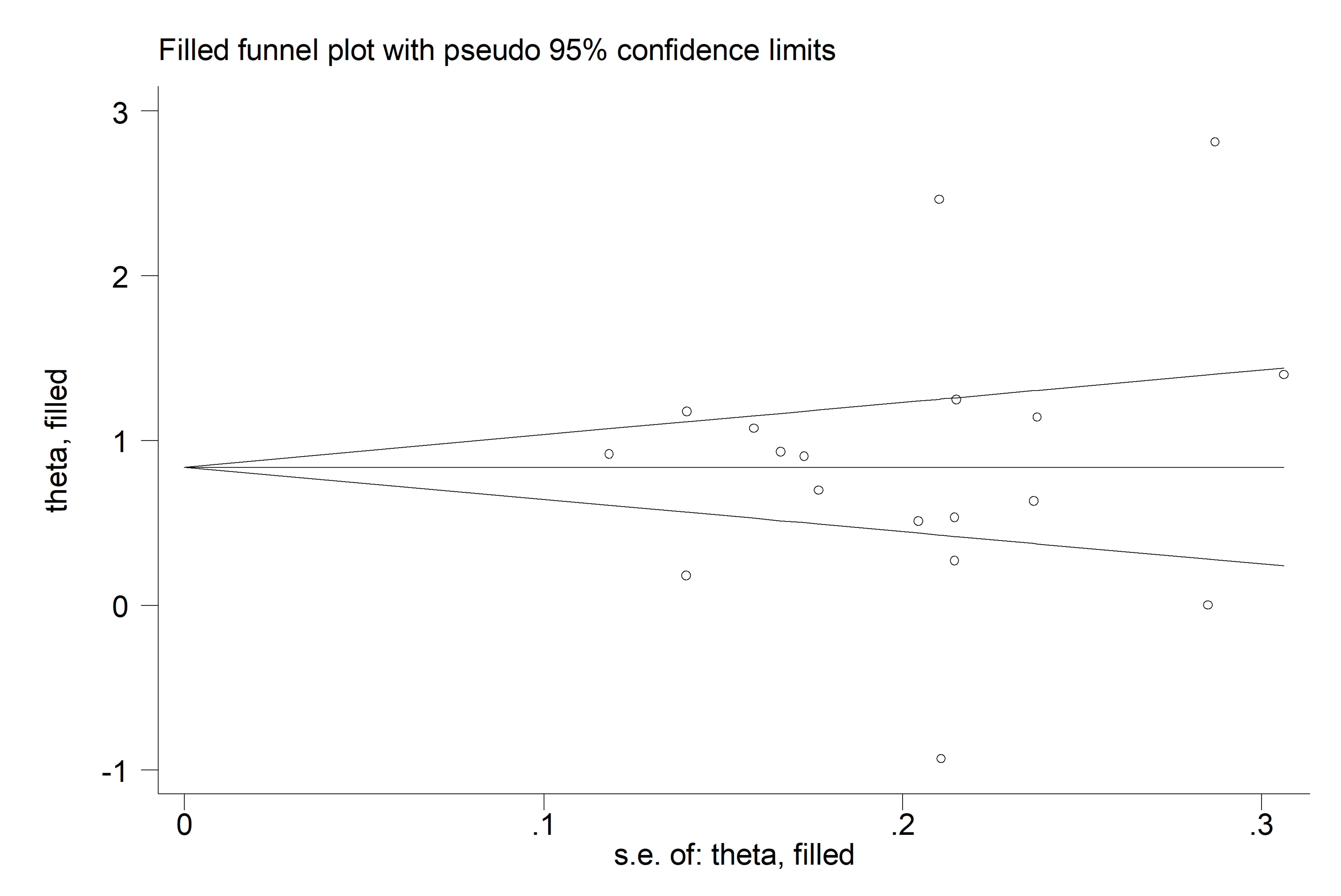

Supplement: Supplementary Figure 8 — Funnel plot of studies investigating the association between the kynurenine/tryptophan ratio and rheumatic disease after “trimming-and-filling”. [file Image_8.tif]

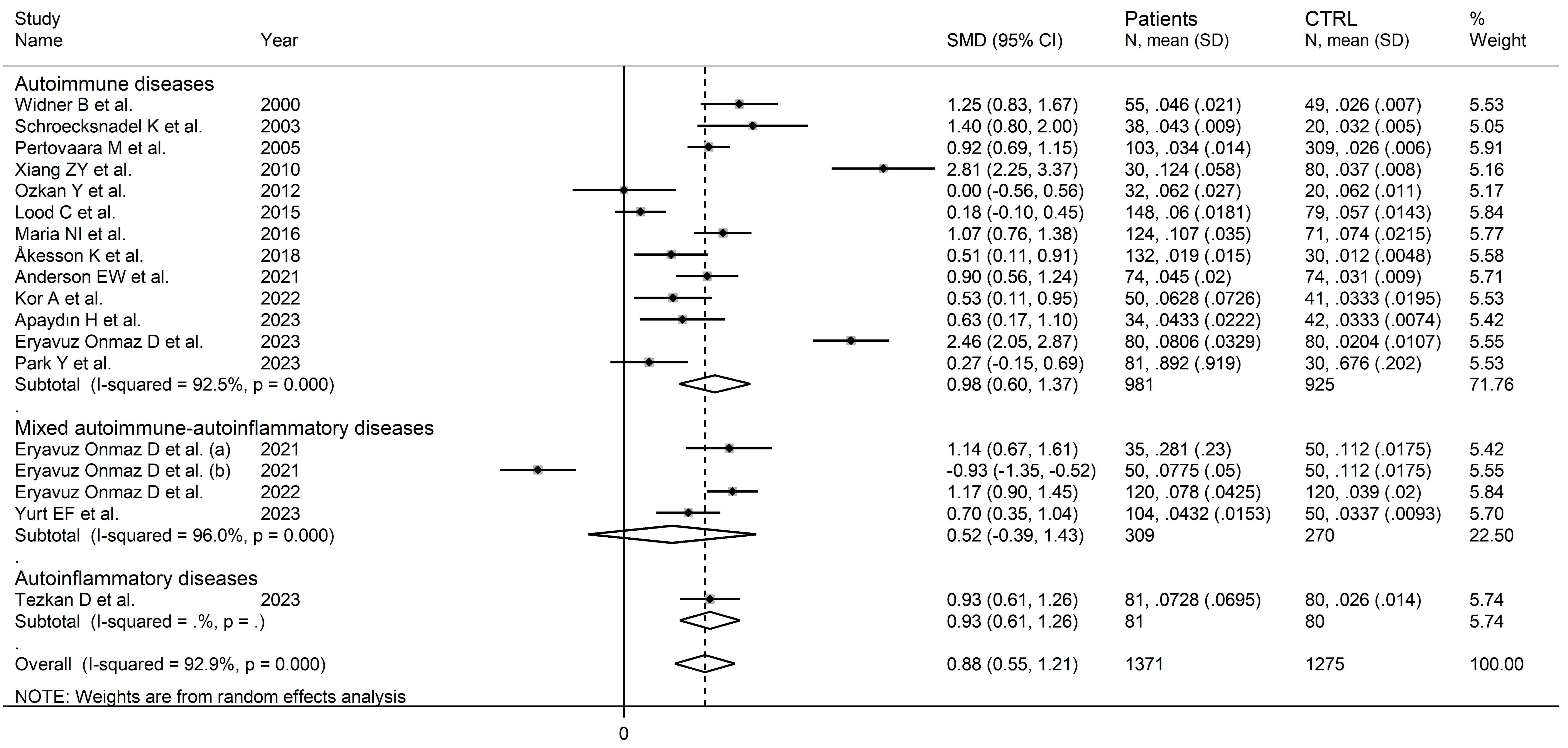

Supplement: Supplementary Figure 9 — Forest plot of studies reporting the kynurenine/tryptophan ratio in patients and controls according to the presence of autoimmune, mixed autoimmune-autoinflammatory, or autoinflammatory diseases. [file Image_9.tif]

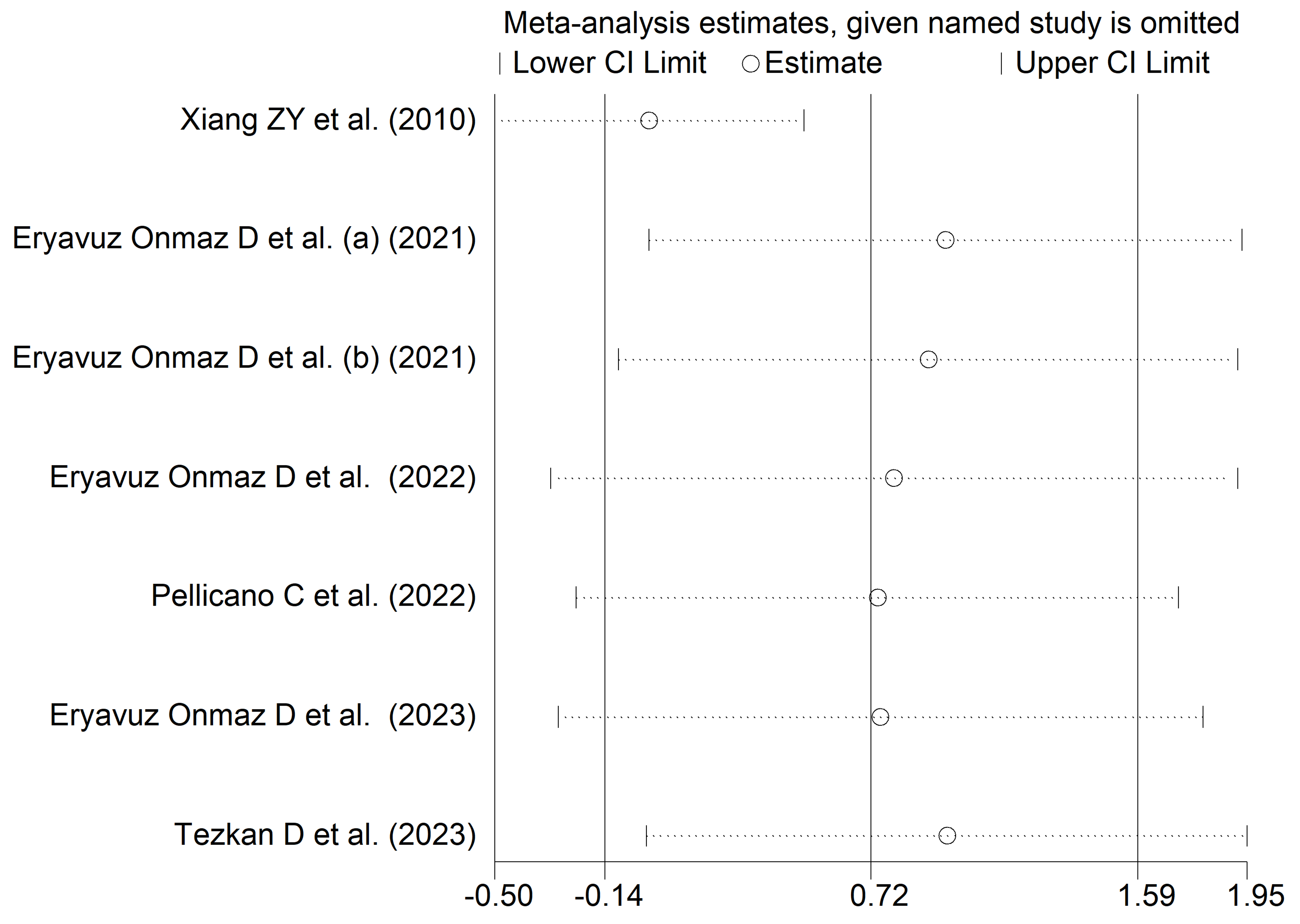

Supplement: Supplementary Figure 10 — Sensitivity analysis of the association between kynurenic acid concentrations and rheumatic disease. [file Image_10.tif]

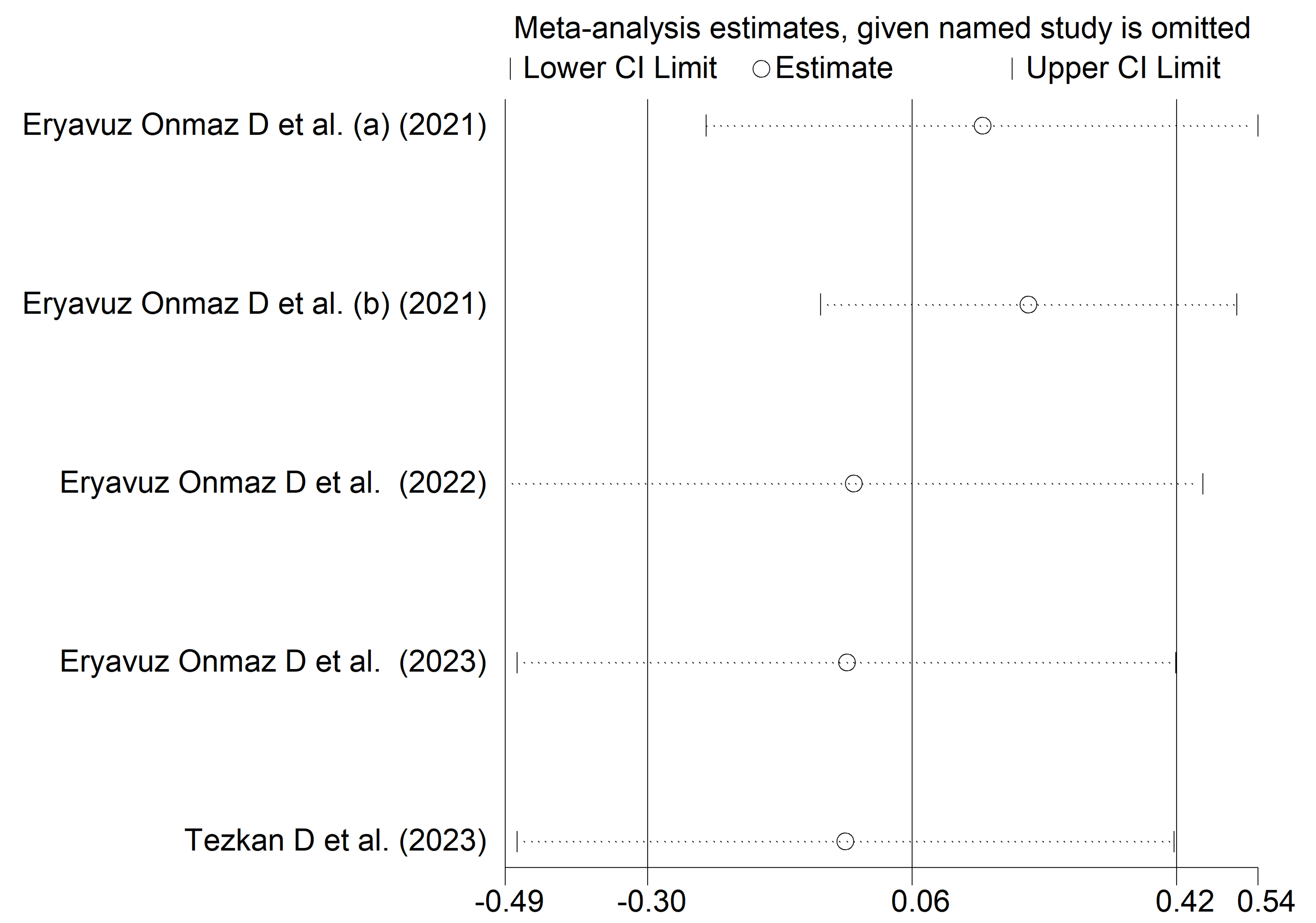

Supplement: Supplementary Figure 11 — Sensitivity analysis of the association between 3-hydroxyanthranilic acid concentrations and rheumatic disease. [file Image_11.tif]

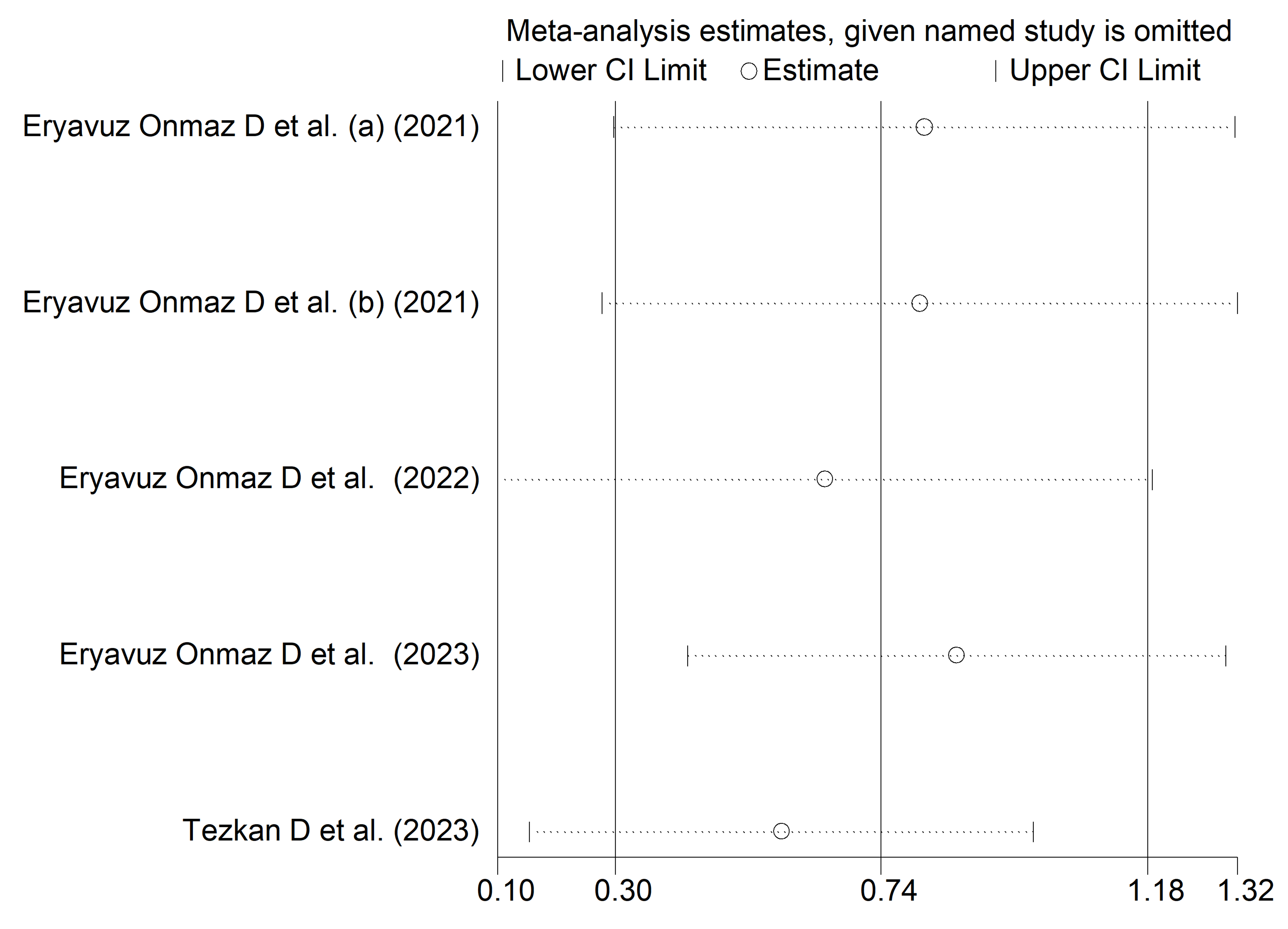

Supplement: Supplementary Figure 12 — Sensitivity analysis of the association between 3-hydroxykynurenine concentrations and rheumatic disease. [file Image_12.tif]

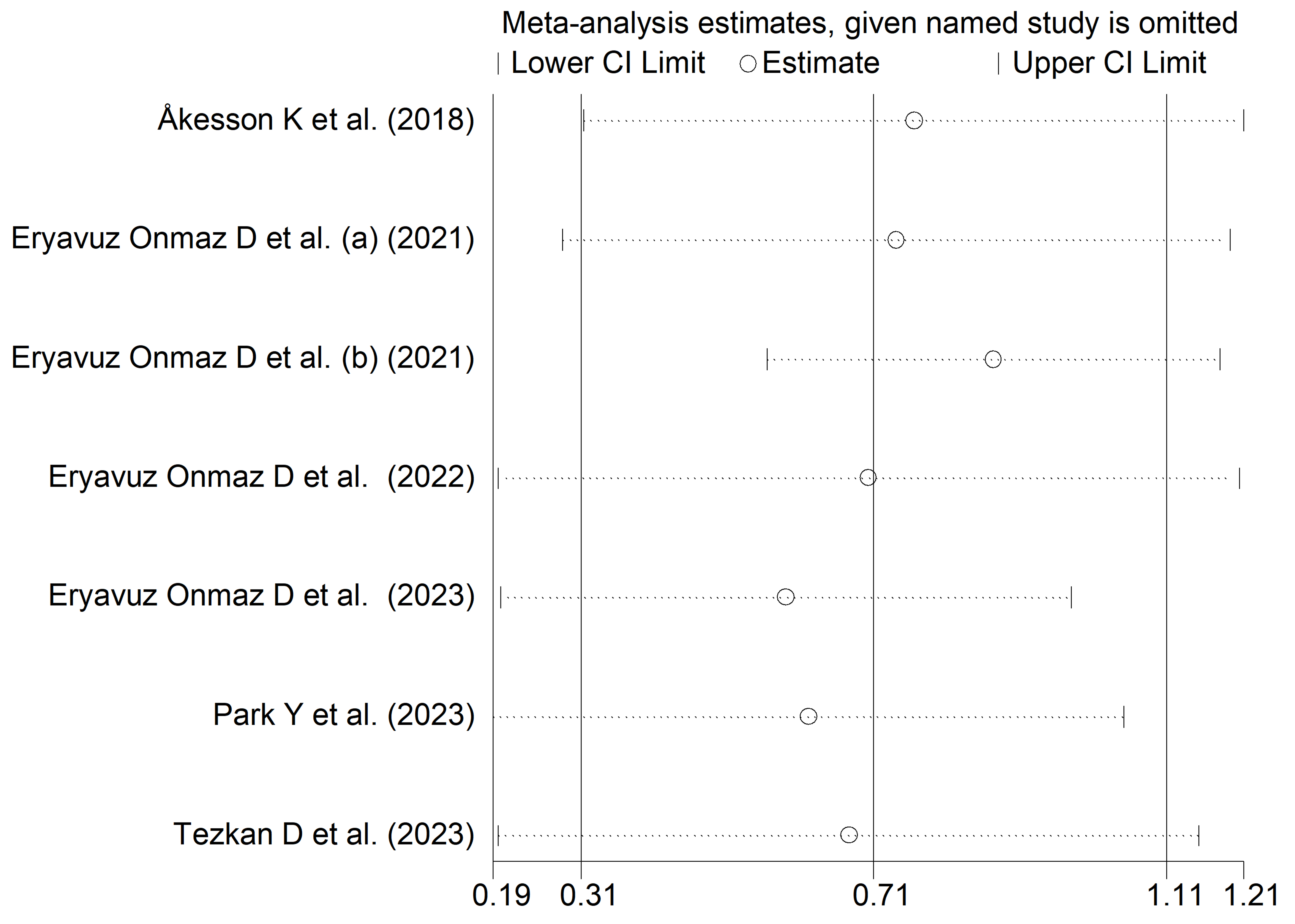

Supplement: Supplementary Figure 13 — Sensitivity analysis of the association between quinolinic acid concentrations and rheumatic disease. [file Image_13.tif]
