# Supplementary material for: A systematic review and meta-analysis of the kynurenine pathway of tryptophan metabolism in rheumatic diseases
Source: Front Immunol. 2023 Oct 23;14:1257159. doi: 10.3389/fimmu.2023.1257159 (PMC10626995; doi:10.3389/fimmu.2023.1257159)
Supplement: Supplementary file 14 [file DataSheet_1.docx]

| **Supplementary Table 1: PRISMA 2020 for abstracts checklist** | | | |
| --- | --- | --- | --- |
| **Section and Topic** | **Item #** | **Checklist item** | **Reported (Yes/No)** |
| **TITLE** | | |  |
| Title | 1 | Identify the report as a systematic review. | Yes |
| **BACKGROUND** | | |  |
| Objectives | 2 | Provide an explicit statement of the main objective(s) or question(s) the review addresses. | Yes |
| **METHODS** | | |  |
| Eligibility criteria | 3 | Specify the inclusion and exclusion criteria for the review. | No (space constraints) |
| Information sources | 4 | Specify the information sources (e.g. databases, registers) used to identify studies and the date when each was last searched. | Yes |
| Risk of bias | 5 | Specify the methods used to assess risk of bias in the included studies. | Yes |
| Synthesis of results | 6 | Specify the methods used to present and synthesise results. | No (space constraints) |
| **RESULTS** | | |  |
| Included studies | 7 | Give the total number of included studies and participants and summarise relevant characteristics of studies. | Yes |
| Synthesis of results | 8 | Present results for main outcomes, preferably indicating the number of included studies and participants for each. If meta-analysis was done, report the summary estimate and confidence/credible interval. If comparing groups, indicate the direction of the effect (i.e. which group is favoured). | Yes |
| **DISCUSSION** | | |  |
| Limitations of evidence | 9 | Provide a brief summary of the limitations of the evidence included in the review (e.g. study risk of bias, inconsistency and imprecision). | Yes |
| Interpretation | 10 | Provide a general interpretation of the results and important implications. | Yes |
| **OTHER** | | |  |
| Funding | 11 | Specify the primary source of funding for the review. | NA |
| Registration | 12 | Provide the register name and registration number. | Yes |

*From:*  Page MJ, McKenzie JE, Bossuyt PM, Boutron I, Hoffmann TC, Mulrow CD, et al. The PRISMA 2020 statement: an updated guideline for reporting systematic reviews. BMJ 2021;372:n71. doi: 10.1136/bmj.n71

For more information, visit: <http://www.prisma-statement.org/>

| **Supplementary Table 2: PRISMA 2020 checklist** | | | |
| --- | --- | --- | --- |
| **Section and Topic** | **Item #** | **Checklist item** | **Location where item is reported** |
| **TITLE** | | |  |
| Title | 1 | Identify the report as a systematic review. | Page 1 |
| **ABSTRACT** | | |  |
| Abstract | 2 | See the PRISMA 2020 for Abstracts checklist. | Page 1 |
| **INTRODUCTION** | | |  |
| Rationale | 3 | Describe the rationale for the review in the context of existing knowledge. | Page 2 |
| Objectives | 4 | Provide an explicit statement of the objective(s) or question(s) the review addresses. | Page 2 |
| **METHODS** | | |  |
| Eligibility criteria | 5 | Specify the inclusion and exclusion criteria for the review and how studies were grouped for the syntheses. | Pages 2-3 |
| Information sources | 6 | Specify all databases, registers, websites, organisations, reference lists and other sources searched or consulted to identify studies. Specify the date when each source was last searched or consulted. | Page 2 |
| Search strategy | 7 | Present the full search strategies for all databases, registers and websites, including any filters and limits used. | See last page of this document |
| Selection process | 8 | Specify the methods used to decide whether a study met the inclusion criteria of the review, including how many reviewers screened each record and each report retrieved, whether they worked independently, and if applicable, details of automation tools used in the process. | Pages 2-3 |
| Data collection process | 9 | Specify the methods used to collect data from reports, including how many reviewers collected data from each report, whether they worked independently, any processes for obtaining or confirming data from study investigators, and details of automation tools used in the process. | Pages 2-3 |
| Data items | 10a | List and define all outcomes for which data were sought. Specify whether all results that were compatible with each outcome domain in each study were sought (e.g. for all measures, time points, analyses), and if not, the methods used to decide which results to collect. | Page 3 |
|  | 10b | List and define all other variables for which data were sought (e.g. participant and intervention characteristics, funding sources). Describe any assumptions made about any missing or unclear information. | Page 3 |
| Study risk of bias assessment | 11 | Specify the methods used to assess risk of bias in the included studies, including details of the tool(s) used, how many reviewers assessed each study and whether they worked independently, and if applicable, details of automation tools used in the process. | Page 3 |
| Effect measures | 12 | Specify for each outcome the effect measure(s) (e.g. risk ratio, mean difference) used in the synthesis or presentation of results. | Page 3 |
| Synthesis methods | 13a | Describe the processes used to decide which studies were eligible for each synthesis (e.g. tabulating the study intervention characteristics and comparing against the planned groups for each synthesis (item #5)). | Page 3 |
|  | 13b | Describe any methods required to prepare the data for presentation or synthesis, such as data conversions. | Page 3 |
|  | 13c | Describe any methods used to tabulate or visually display results of individual studies and syntheses. | Page 3 |
|  | 13d | Describe any methods used to synthesize results and provide a rationale for the choice(s). If meta-analysis was performed, describe the model(s), method(s) to identify the presence and extent of statistical heterogeneity, and software package(s) used. | Page 3 |
|  | 13e | Describe any methods used to explore possible causes of heterogeneity among study results (e.g. subgroup analysis, meta-regression). | Page 3 |
|  | 13f | Describe any sensitivity analyses conducted to assess robustness of the synthesized results. | Page 3 |
| Reporting bias assessment | 14 | Describe any methods used to assess risk of bias due to missing results in a synthesis (arising from reporting biases). | Page 3 |
| Certainty assessment | 15 | Describe any methods used to assess certainty (or confidence) in the body of evidence for an outcome. | Page 3 |
| **RESULTS** | | |  |
| Study selection | 16a | Describe the results of the search and selection process, from the number of records identified in the search to the number of studies included in the review, ideally using a flow diagram. | Figure 2 |
|  | 16b | Cite studies that might appear to meet the inclusion criteria, but which were excluded, and explain why they were excluded. | Figure 2 |
| Study characteristics | 17 | Cite each included study and present its characteristics. | Table 1 |
| Risk of bias in studies | 18 | Present assessments of risk of bias for each included study. | Supplementary Table 3 |
| Results of individual studies | 19 | For all outcomes, present, for each study: (a) summary statistics for each group (where appropriate) and (b) an effect estimate and its precision (e.g. confidence/credible interval), ideally using structured tables or plots. | Table 1 |
| Results of syntheses | 20a | For each synthesis, briefly summarise the characteristics and risk of bias among contributing studies. | Pages 3-10 |
|  | 20b | Present results of all statistical syntheses conducted. If meta-analysis was done, present for each the summary estimate and its precision (e.g. confidence/credible interval) and measures of statistical heterogeneity. If comparing groups, describe the direction of the effect. | Figures 3-20 |
|  | 20c | Present results of all investigations of possible causes of heterogeneity among study results. | Pages 3-10 |
|  | 20d | Present results of all sensitivity analyses conducted to assess the robustness of the synthesized results. | Pages 3-10 |
| Reporting biases | 21 | Present assessments of risk of bias due to missing results (arising from reporting biases) for each synthesis assessed. | Pages 3-10 |
| Certainty of evidence | 22 | Present assessments of certainty (or confidence) in the body of evidence for each outcome assessed. | Pages 3-10 |
| **DISCUSSION** | | |  |
| Discussion | 23a | Provide a general interpretation of the results in the context of other evidence. | Pages 10-11 |
|  | 23b | Discuss any limitations of the evidence included in the review. | Pages 10-11 |
|  | 23c | Discuss any limitations of the review processes used. | Pages 10-11 |
|  | 23d | Discuss implications of the results for practice, policy, and future research. | Pages 10-11 |
| **OTHER INFORMATION** | | |  |
| Registration and protocol | 24a | Provide registration information for the review, including register name and registration number, or state that the review was not registered. | Pages 1, 3 |
|  | 24b | Indicate where the review protocol can be accessed, or state that a protocol was not prepared. | Pages 1, 3 |
|  | 24c | Describe and explain any amendments to information provided at registration or in the protocol. | Page 12 |
| Support | 25 | Describe sources of financial or non-financial support for the review, and the role of the funders or sponsors in the review. | NA |
| Competing interests | 26 | Declare any competing interests of review authors. | Page 12 |
| Availability of data | 27 | Report which of the following are publicly available and where they can be found: template data collection forms; data extracted from included studies; data used for all analyses; analytic code; any other materials used in the review. | Page 12 |

*From:*  Page MJ, McKenzie JE, Bossuyt PM, Boutron I, Hoffmann TC, Mulrow CD, et al. The PRISMA 2020 statement: an updated guideline for reporting systematic reviews. BMJ 2021;372:n71. doi: 10.1136/bmj.n71

For more information, visit: <http://www.prisma-statement.org/>

**Search strategy**

**PubMed**

(“tryptophan”[All Fields] OR “kynuren*”[All Fields] OR “anthranil*”[All Fields] OR “xanthurenic”[All Fields] OR “cinnabar*”[All Fields] OR “picolinic”[All Fields] OR “quinolinic”[All Fields]) AND (“rheumatic diseases”[All Fields] OR “rheumatoid arthritis”[All Fields] OR “psoriatic arthritis”[All Fields] OR “ankylosing spondylitis”[All Fields] OR “systemic lupus erythematosus”[All Fields] OR “systemic sclerosis”[All Fields] OR “Sjogren's syndrome”[All Fields] OR “connective tissue diseases”[All Fields] OR “vasculitis”[All Fields] OR “Behcet's disease”[All Fields])

**Web of Science**

“tryptophan” OR “kynuren*” OR “anthranil*” OR “xanthurenic” OR “cinnabar*” OR “picolinic” OR “quinolinic” (All Fields) AND “rheumatic diseases” OR “rheumatoid arthritis” OR “psoriatic arthritis” OR “ankylosing spondylitis” OR “systemic lupus erythematosus” OR “systemic sclerosis” OR “Sjogren’s syndrome” OR “connective tissue diseases” OR “vasculitis” OR “Behçet’s disease” (All Fields)

**Scopus**

(TITLE-ABS-KEY (“tryptophan” OR “kynuren*” OR “anthranil*” OR “xanthurenic” OR “cinnabar*” OR “picolinic” OR “quinolinic”) AND TITLE-ABS-KEY (“rheumatic diseases” OR “rheumatoid arthritis” OR “psoriatic arthritis” OR “ankylosing spondylitis” OR “systemic lupus erythematosus” OR “systemic sclerosis” OR “Sjogren’s syndrome” OR “connective tissue diseases” OR “vasculitis” OR “Behçet’s disease”

**Supplementary Table 3.** The Joanna Briggs Institute critical appraisal checklist.

| **Study** | **Were the inclusion criteria clearly defined?** | **Were the subjects and the setting described in detail?** | **Was the exposure measured in a valid and reliable way?** | **Were objective, standard criteria used for measurement of the condition?** | **Were confounding factors identified?** | **Were strategies to deal with confounding factors stated?** | **Were the outcomes measured in a valid and reliable way?** | **Was appropriate statistical analysis used?** | **Risk of bias** |
| --- | --- | --- | --- | --- | --- | --- | --- | --- | --- |
| Csipo et al. (1) | Yes | No | Yes | Yes | No | No | Yes | Yes | Moderate |
| Widner et al. (2) | No | Yes | Yes | Yes | No | No | Yes | Yes | Moderate |
| Schroecksnadel et al. (3) | No | Yes | Yes | Yes | No | No | Yes | Yes | Moderate |
| Pertovaara et al. (4) | No | Yes | Yes | Yes | No | No | Yes | Yes | Moderate |
| Xiang et al. (5) | No | Yes | Yes | Yes | No | No | Yes | Yes | Moderate |
| Ozkan et al. (6) | No | Yes | Yes | Yes | No | No | Yes | Yes | Moderate |
| Lood et al. (7) | No | Yes | Yes | Yes | No | No | Yes | Yes | Moderate |
| Maria et al. (8) | No | Yes | Yes | Yes | No | No | Yes | Yes | Moderate |
| Smolenska et al. (9) | Yes | Yes | Yes | Yes | No | No | Yes | Yes | Low |
| Åkesson et al. (10) | No | Yes | Yes | Yes | No | No | Yes | Yes | Moderate |
| Urbaniak et al. (11) | Yes | Yes | Yes | Yes | No | No | Yes | Yes | Low |
| Smolenska et al. (12) | No | Yes | Yes | Yes | No | No | Yes | Yes | Moderate |
| Zhou et al. (13) | Yes | Yes | Yes | Yes | Yes | Yes | Yes | Yes | Low |
| Anderson et al. (14) | Yes | Yes | Yes | Yes | Yes | Yes | Yes | Yes | Low |
| Eryavuz Onmaz et al. (15) | Yes | Yes | Yes | Yes | No | No | Yes | Yes | Low |
| Kor et al. (16) | No | Yes | Yes | Yes | No | No | Yes | Yes | Moderate |
| Eryavuz Onmaz et al. (17) | Yes | Yes | Yes | Yes | No | No | Yes | Yes | Low |
| Pellicano et al. (18) | Yes | Yes | Yes | Yes | Yes | Yes | Yes | Yes | Low |
| Apaydın et al. (19) | No | Yes | Yes | Yes | No | No | Yes | Yes | Moderate |
| Jeon et al. (20) | No | Yes | Yes | Yes | No | No | Yes | Yes | Moderate |
| Eryavuz Onmaz et al. (21) | Yes | Yes | Yes | Yes | Yes | Yes | Yes | Yes | Low |
| Park et al. (22) | No | Yes | Yes | Yes | No | No | Yes | Yes | Moderate |
| Tezkan et al (23) | Yes | Yes | Yes | Yes | No | No | Yes | Yes | Low |
| Yurt et al. (24) | Yes | Yes | Yes | Yes | No | No | Yes | Yes | Low |

**References**

1. Csipo I, Czirjak L, Szanto S, Szerafin L, Sipka S, Szegedi G. Decreased serum tryptophan and elevated neopterin levels in systemic sclerosis. Clin Exp Rheumatol. 1995;13(2):269-70. doi:

2. Widner B, Sepp N, Kowald E, Ortner U, Wirleitner B, Fritsch P, et al. Enhanced tryptophan degradation in systemic lupus erythematosus. Immunobiology. 2000;201(5):621-30. doi: 10.1016/S0171-2985(00)80079-0

3. Schroecksnadel K, Kaser S, Ledochowski M, Neurauter G, Mur E, Herold M, et al. Increased degradation of tryptophan in blood of patients with rheumatoid arthritis. J Rheumatol. 2003;30(9):1935-9. doi:

4. Pertovaara M, Raitala A, Uusitalo H, Pukander J, Helin H, Oja SS, et al. Mechanisms dependent on tryptophan catabolism regulate immune responses in primary Sjogren's syndrome. Clin Exp Immunol. 2005;142(1):155-61. doi: 10.1111/j.1365-2249.2005.02889.x

5. Xiang ZY, Tang AG, Ren YP, Zhou QX, Luo XB. Simultaneous determination of serum tryptophan metabolites in patients with systemic lupus erythematosus by high performance liquid chromatography with fluorescence detection. Clin Chem Lab Med. 2010;48(4):513-7. doi: 10.1515/CCLM.2010.105

6. Ozkan Y, Mete G, Sepici-Dincel A, Sepici V, Simsek B. Tryptophan degradation and neopterin levels in treated rheumatoid arthritis patients. Clin Rheumatol. 2012;31(1):29-34. doi: 10.1007/s10067-011-1767-5

7. Lood C, Tyden H, Gullstrand B, Klint C, Wenglen C, Nielsen CT, et al. Type I interferon-mediated skewing of the serotonin synthesis is associated with severe disease in systemic lupus erythematosus. PLoS One. 2015;10(4):e0125109. doi: 10.1371/journal.pone.0125109

8. Maria NI, van Helden-Meeuwsen CG, Brkic Z, Paulissen SM, Steenwijk EC, Dalm VA, et al. Association of Increased Treg Cell Levels With Elevated Indoleamine 2,3-Dioxygenase Activity and an Imbalanced Kynurenine Pathway in Interferon-Positive Primary Sjogren's Syndrome. Arthritis Rheumatol. 2016;68(7):1688-99. doi: 10.1002/art.39629

9. Smolenska Z, Smolenski RT, Zdrojewski Z. Plasma concentrations of amino acid and nicotinamide metabolites in rheumatoid arthritis--potential biomarkers of disease activity and drug treatment. Biomarkers. 2016;21(3):218-24. doi: 10.3109/1354750X.2015.1130746

10. Akesson K, Pettersson S, Stahl S, Surowiec I, Hedenstrom M, Eketjall S, et al. Kynurenine pathway is altered in patients with SLE and associated with severe fatigue. Lupus Sci Med. 2018;5(1):e000254. doi: 10.1136/lupus-2017-000254

11. Urbaniak B, Plewa S, Klupczynska A, Sikorska D, Samborski W, Kokot ZJ. Serum free amino acid levels in rheumatoid arthritis according to therapy and physical disability. Cytokine. 2019;113:332-9. doi: 10.1016/j.cyto.2018.10.002

12. Smolenska Z, Zabielska-Kaczorowska M, Wojteczek A, Kutryb-Zajac B, Zdrojewski Z. Metabolic Pattern of Systemic Sclerosis: Association of Changes in Plasma Concentrations of Amino Acid-Related Compounds With Disease Presentation. Front Mol Biosci. 2020;7:585161. doi: 10.3389/fmolb.2020.585161

13. Zhou Y, Zhang X, Chen R, Han S, Liu Y, Liu X, et al. Serum amino acid metabolic profiles of ankylosing spondylitis by targeted metabolomics analysis. Clin Rheumatol. 2020;39(8):2325-36. doi: 10.1007/s10067-020-04974-z

14. Anderson EW, Fishbein J, Hong J, Roeser J, Furie RA, Aranow C, et al. Quinolinic acid, a kynurenine/tryptophan pathway metabolite, associates with impaired cognitive test performance in systemic lupus erythematosus. Lupus Sci Med. 2021;8(1). doi: 10.1136/lupus-2021-000559

15. Eryavuz Onmaz D, Sivrikaya A, Isik K, Abusoglu S, Albayrak Gezer I, Humeyra Yerlikaya F, et al. Altered kynurenine pathway metabolism in patients with ankylosing spondylitis. Int Immunopharmacol. 2021;99:108018. doi: 10.1016/j.intimp.2021.108018

16. Kor A, Erten Ş, Yurt EF, Dogan İ, Apaydin H, Aserdar M, et al. Clinical significance of plasma tryptophan, kynurenine, and kynurenine/tryptophan ratio in rheumatoid arthritis patients. The Egyptian Rheumatologist. 2022;44(4):367-71. doi: 10.1016/j.ejr.2022.07.005

17. Eryavuz Onmaz D, Tezcan D, Abusoglu S, Sivrikaya A, Kuzu M, Yerlikaya FH, et al. Elevated serum levels of kynurenine pathway metabolites in patients with Behcet disease. Amino Acids. 2022;54(6):877-87. doi: 10.1007/s00726-022-03170-4

18. Pellicano C, Vaiarello V, Colalillo A, Gigante A, Iannazzo F, Rosato E. Role of kinurenic acid in the systemic sclerosis renal involvement. Clin Exp Med. 2022. doi: 10.1007/s10238-022-00962-6

19. Apaydin H, Koca Bicer C, Feyza Yurt E, Abdulkadir Serdar M, Dogan I, Erten S. Elevated Kynurenine Levels in Patients with Primary Sjogren's Syndrome. Lab Med. 2023;54(2):166-72. doi: 10.1093/labmed/lmac084

20. Jeon C, Jang Y, Lee SH, Weon S, Park H, Lee S, et al. Abnormal kynurenine level contributes to the pathological bone features of ankylosing spondylitis. Int Immunopharmacol. 2023;118:110132. doi: 10.1016/j.intimp.2023.110132

21. Eryavuz Onmaz D, Tezcan D, Abusoglu S, Sak F, Humeyra Yerlikaya F, Yilmaz S, et al. Impaired kynurenine metabolism in patients with primary Sjogren's syndrome. Clin Biochem. 2023;114:1-10. doi: 10.1016/j.clinbiochem.2023.01.007

22. Park Y, Lee JJ, Koh JH, Kim MJ, Park SH, Kwok SK. Kynurenine pathway can be a potential biomarker of fatigue in primary Sjogren's syndrome. Clin Exp Rheumatol. 2023. doi: 10.55563/clinexprheumatol/cp4st9

23. Tezcan D, Onmaz DE, Sivrikaya A, Korez MK, Hakbilen S, Gulcemal S, et al. Kynurenine pathway of tryptophan metabolism in patients with familial Mediterranean fever. Mod Rheumatol. 2023;33(2):398-407. doi: 10.1093/mr/roac016

24. Yurt EF, Bicer C, Serdar MA, Akan S, Erten S. Accelerated kynurenine pathway downregulates immune activation in patients with axial spondyloarthritis. Cytokine. 2023;169:156247. doi: 10.1016/j.cyto.2023.156247
